# REDD1 loss reprograms lipid metabolism to drive progression of *RAS* mutant tumors

**DOI:** 10.1101/gad.335166.119

**Published:** 2020-06-01

**Authors:** Shuxi Qiao, Siang-Boon Koh, Varunika Vivekanandan, Devika Salunke, Krushna Chandra Patra, Elma Zaganjor, Kenneth Ross, Yusuke Mizukami, Sarah Jeanfavre, Athena Chen, Mari Mino-Kenudson, Sridhar Ramaswamy, Clary Clish, Marcia Haigis, Nabeel Bardeesy, Leif W. Ellisen

**Affiliations:** 1 Massachusetts General Hospital Cancer Center, Boston, Massachusetts 02114, USA;; 2 Harvard Medical School, Boston, Massachusetts 02115, USA;; 3 Ludwig Cancer Center at Harvard, Harvard University, Boston, Massachusetts 02115, USA;; 4 Broad Institute of Massachusetts Institute of Technology and Harvard University, Cambridge, Massachusetts 02142, USA;; 5 Department of Pathology, Massachusetts General Hospital, Massachusetts 02114, USA

**Keywords:** RAS, REDD1, metastasis, lipid metabolism, oxidative stress, energy stress, lysophospholipids, fatty acid oxidation, glycolysis

## Abstract

Human cancers with activating *RAS* mutations are typically highly aggressive and treatment-refractory, yet *RAS* mutation itself is insufficient for tumorigenesis, due in part to profound metabolic stress induced by RAS activation. Here we show that loss of REDD1, a stress-induced metabolic regulator, is sufficient to reprogram lipid metabolism and drive progression of *RAS* mutant cancers. *Redd1* deletion in genetically engineered mouse models (GEMMs) of KRAS-dependent pancreatic and lung adenocarcinomas converts preneoplastic lesions into invasive and metastatic carcinomas. Metabolic profiling reveals that REDD1-deficient/*RAS* mutant cells exhibit enhanced uptake of lysophospholipids and lipid storage, coupled to augmented fatty acid oxidation that sustains both ATP levels and ROS-detoxifying NADPH. Mechanistically, REDD1 loss triggers HIF-dependent activation of a lipid storage pathway involving PPARγ and the prometastatic factor CD36. Correspondingly, decreased REDD1 expression and a signature of REDD1 loss predict poor outcomes selectively in *RAS* mutant but not *RAS* wild-type human lung and pancreas carcinomas. Collectively, our findings reveal the REDD1-mediated stress response as a novel tumor suppressor whose loss defines a *RAS* mutant tumor subset characterized by reprogramming of lipid metabolism, invasive and metastatic progression, and poor prognosis. This work thus provides new mechanistic and clinically relevant insights into the phenotypic heterogeneity and metabolic rewiring that underlies these common cancers.

## Abstract (precis)

In this study, Qiao et al. set out to investigate the role of REDD1 in the development of KRAS-driven tumors. Using genetically engineered mouse models, the authors show that loss of REDD1 promotes the development of oncogenic KRAS-driven pancreatic and lung cancers. Additionally, the authors use a combination of transcriptomic and metabolomic analyses to show that REDD1 deficiency induces lipid uptake, enhances fatty acid oxidation, and suppresses de novo lipid biosynthesis, in particular under hypoxia conditions, which plays an important role for the redox homeostasis of tumor cells through the regulation of NADPH levels.

Activating mutations in *RAS* family members are observed in a substantial proportion of human cancers, where they are associated with aggressive behavior and poor clinical outcomes (Pylayeva-Gupta et al. 2011). Despite a relatively detailed understanding of the pathways downstream from RAS activation, selectively targeting these pathways has met with limited clinical success. While this fact relates in part to the myriad downstream effects of RAS, it also reflects the various collateral adaptations that the mutant cells undergo to cope with metabolic stress engendered by RAS activation. Numerous studies in recent years have documented a fundamental reconfiguring of metabolism in the context of *RAS* mutation, including up-regulation of nutrient acquisition pathways, together with rewiring of mechanisms for biosynthesis, energy generation, and detoxification of reactive oxygen species (ROS) (DeNicola et al. 2011; White 2013; Harris et al. 2015). Nonetheless, the observed heterogeneity in the genomic organization and clinical behavior of *RAS* mutant cancers strongly suggests distinct mechanisms of metabolic rewiring in different tumor subsets that remain incompletely characterized.

Pivotal studies on metabolism in the context of activated RAS have revealed altered glucose utilization via aerobic glycolysis, the Warburg effect, which facilitates shunting of glycolytic intermediates into biosynthetic pathways (Ying et al. 2012). This adaptation is accompanied by altered utilization of glutamine, which provides a source of TCA cycle intermediates for oxidative ATP generation and for cytosolic export and subsequent generation of ROS-detoxifying NADPH (Son et al. 2013). Alterations in lipid metabolism in *RAS* mutant tumors have generally received less attention, but recent studies have implicated deregulated lipid synthesis, uptake, storage, and catabolism as potential contributors in this context (Kamphorst et al. 2013; Bensaad et al. 2014; Padanad et al. 2016; Svensson et al. 2016; Patra et al. 2018). Overall, however, the mechanisms and phenotypic consequences of altered lipid metabolism in RAS-driven tumors are poorly understood.

As *RAS* mutation induces profound metabolic stress, endogenous stress response pathways may serve as barriers to RAS-mediated tumor progression (Biancur and Kimmelman 2018). An intriguing potential factor in this regard is REDD1, which is up-regulated in response to hypoxia and energy stress, and functions as a pleiotropic regulator of cell metabolism (Ellisen 2005; Gordon et al. 2016b; Lipina and Hundal 2016). Both mammalian REDD1 and its *Drosophila* orthologs inhibit TORC1 kinase activity in the acute response to hypoxia (Brugarolas et al. 2004; Reiling and Hafen 2004), while genetic and biochemical studies have demonstrated both mTORC1-dependent and TORC1-independent roles for REDD1 in control of glycolysis, autophagy, and mitochondrial oxidative metabolism (DeYoung et al. 2008; Horak et al. 2010; Qiao et al. 2015; Gordon et al. 2016a; Alvarez-Garcia et al. 2017). Phenotypes associated with *REDD1* genetic loss support its role as a physiological mediator of diverse pathologic cellular stress responses. In lung tissue, oxidative stress resulting from chronic cigarette smoke exposure induces REDD1 and results in tissue destruction known as emphysema, and *Redd1*-null mice exhibit protection against smoke-induced emphysema (Yoshida et al. 2010). REDD1 has also been associated with stress responses in the central nervous system (CNS), where REDD1 is activated in response to environmental stress to suppress mTORC1-mediated phosphorylation. *Redd1*-null mice are protected against both of these biochemical changes and the behavioral manifestations of stress-induced depression (Ota et al. 2014). The contribution of REDD1 to human cancer has remained uncertain, as *Redd1*-null mice do not die prematurely or exhibit tumor predisposition, and studies have reported mixed results regarding the contribution of REDD1 to cell proliferation and survival relevant to tumorigenesis (DeYoung et al. 2008; Reuschel et al. 2015; Lipina and Hundal 2016).

Given the overlap and potential interaction of pathways controlled by REDD1 and RAS, we analyzed the phenotypic and metabolic effects of REDD1 loss in the context of RAS activation. While *RAS* mutation alone in lung and pancreatic epithelium induces preneoplastic lesions, loss of REDD1 in *RAS* mutant cells promotes rapidly growing invasive carcinomas and distant metastatic dissemination. Biochemical and metabolic studies reveal that loss of REDD1 activates lipid uptake and fatty acid oxidation to meet the metabolic and energetic demands of RAS activation. Accordingly, in vivo studies demonstrate the vulnerability of these tumors to antioxidant depletion, while analysis of human tumors shows that decreased REDD1 expression predicts poor patient survival selectively in *RAS* mutant lung and pancreas carcinomas. Collectively, our findings reveal that a deregulated REDD1-mediated stress response underpins a previously unidentified, metabolically distinct and poor-prognosis subset of *RAS* mutant cancers.

## Results

### REDD1 deficiency cooperates with mutant KRAS to drive tumor progression

**Figure 1.**
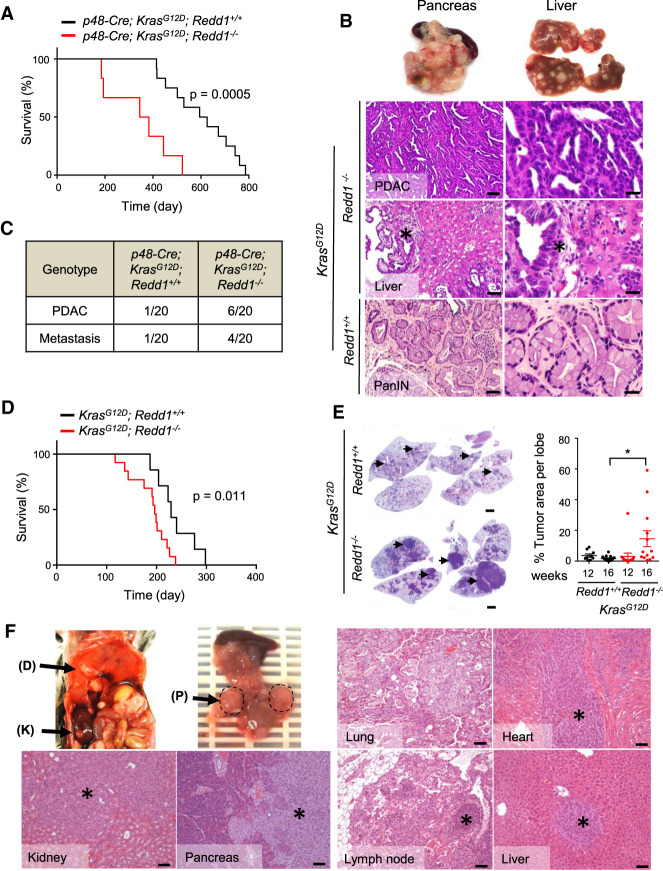
REDD1 deficiency cooperates with KRAS to drive tumor progression and metastatic dissemination. (*A*) Kaplan-Meier analysis of *p48-Cre;Kras^G12D^; Redd1*^−/−^ (*p48KR*) mice (*n* = 6, median survival = 364 d) and *p48-Cre;Kras^G12D^;Redd1*^+/+^ (*p48K*) mice (*n* = 12, median survival = 610.5 d). All animals euthanized exhibited PanIN and/or PDAC. *P* = 0.0005 by log-rank test. (*B*, *top*) representative gross photographs depicting enlarged, nodule-studded pancreas and enlarged spleen (*left*) and liver with metastatic foci (*right*) from 17-mo-old *p48KR* mice. (*Bottom*) Hematoxylin and eosin (H&E)-stained tissue sections from the indicated genotypes. The *right* panels represent higher magnification of the respective *left* panels. *p48KR* mice show PDAC and liver metastasis (indicated by an asterisk) compared with *p48K* mice that show early PanIN histology. Scale bars: *left*, 50 µm; *right* 20 µm. (*C*) Summary of histological findings of PDAC and metastasis including those reported in *A* and additional mice sacrificed at 11 mo. Total *n* = 20 mice analyzed per genotype. (*D*) Kaplan-Meier analysis of *Kras*^G12D^; *Redd1*^+/+^ and *Kras^G12D^; Redd1*^−/−^mice after intratracheal adenoviral Cre (*AdK* and *AdKR* mice, respectively). *AdK* mice (median survival = 229 d, *n* = 7); *AdKR* mice, median survival = 196 d, *n* = 13. Survival times are after infection. *P* = 0.011 by log-rank test. (*E*, *left*) Representative H&E-stained sections of whole-mount lung tissues from a pair of age- and sex-matched *AdK* and *AdKR* mice 20 wk after intratracheal delivery of Cre. Tumors are indicated by arrows. Scale bars, 2 mm. (*Right*) Quantification of lung tumor burden per lung lobe from mice of the denoted genotypes. Each dot indicates one lobe (*n* ≥ 2 mice for each time point per genotype). Horizontal lines indicate mean ± SEM. (*) *P* = 0.01, by two-tailed *t*-test. (*F*) Necropsy of *AdKR* mice euthanized 28 wk after intratracheal Cre treatment showing metastasis to kidney (K), diaphragm (D), and pancreas (P). H&E-stained sections of primary lung tumor and metastatic foci (indicated by an asterisk) are shown. Scale bars, 100 µm. See also Supplemental Figure S1.

To test the hypothesis that REDD1 may function as a barrier to RAS-driven tumorigenesis in vivo we created GEMMs, intercrossing *Redd1*^−/−^ mice with the inducible knock-in *Ras* mutant allele *loxP-stop-loxP* (*LSL*) *Kras*^*G12D*^ (*Kras^G12D/+^*) (Supplemental Fig. S1A; Jackson et al. 2001; Sofer et al. 2005). Consistent with previous reports, we found that in B6 mice, crossing *LSL-Kras^G12D/+^* alone to the pancreas-specific Cre recombinase allele *p48-Cre* (hereafter *p48K* mice) results in preneoplastic lesions known as pancreatic intraepithelial neoplasm (PanIN) and a median survival (to a humane endpoint) of ∼2 yr, but only rarely invasive PDAC (Fig. 1A; Hingorani et al. 2003). These mice ultimately succumb to sequelae of pancreatic insufficiency related to extensive PanIN (Hingorani et al. 2003). In contrast, a matched cohort of mice with loss of REDD1 (*p48-Cre*;*LSL-Kras^G12D/+^;Redd1*^−/−^; hereafter *p48KR*) had dramatically shortened survival (Fig. 1A; Supplemental Fig. S1B). These mice were universally found to have extensive PanIN, and a nearly one-third had already progressed to invasive PDAC (Fig. 1B,C; Supplemental Fig. S1C). Notably, the majority of PDAC-bearing *p48KR* mice exhibited extensive metastatic disease, including to liver and lung. In contrast, both invasive PDAC and metastasis were rare in *p48K* mice (Fig. 1C; Supplemental Fig. S1B).

We wished to determine whether this phenotype is likely to result from a cell-autonomous effect of REDD1 loss. Thus, we isolated primary *p48-Cre*; *LSL-Kras^G12D/+^* pancreatic epithelial cells (hereafter, KPECs), then ablated REDD1 expression via lentiviral shRNA ex vivo and reimplanted cells into the pancreas of immunodeficient hosts (Supplemental Fig. S1D; Corcoran et al. 2011). At 4 wk after implantation, vector-expressing KPECs formed small masses of well-differentiated neoplasms, whereas the matched cells expressing REDD1 shRNA developed significantly larger, invasive tumors demonstrating histological features consistent with poorly differentiated PDAC (Supplemental Fig. S1E,F). Thus, loss of REDD1 confers a cell-autonomous advantage to *Kras* mutant pancreatic epithelia in vivo, resulting in aggressive, invasive tumor progression.

We then developed a second model to establish whether the cooperative effect of REDD1 loss on RAS-dependent tumorigenesis was limited to the PDAC context. Thus, we exposed *LSL-Kras^G12D/+^* mice to intratracheal adenoviral Cre (Ad-Cre) (Supplemental Fig. S1A). As demonstrated previously, such mice develop multifocal pulmonary adenomas that rarely progress to invasive adenocarcinomas, although these mice ultimately die of pulmonary complications (Fig. 1D,E; Jackson et al. 2001). However, when we compared *LSL-Kras^G12D/+^*; *Redd1*^+/+^ and *LSL-Kras^G12D/+^*; *Redd1*^−/−^ mice exposed to Ad-Cre (hereafter *AdK* and *AdKR* mice, respectively), we noted significantly shorter survival for the *AdKR* mice (Fig. 1D). Necropsy revealed substantially greater tumor burden in the lungs of *AdKR* mice, including frequent progression to invasive adenocarcinomas (Fig. 1E,F; Supplemental Fig. S1G). Furthermore, a subset of *AdKR* mice exhibited grossly evident hematogenous metastasis to distant organs including liver and kidney (Fig. 1F; Supplemental Fig. S1H). Collectively, these findings suggest that loss of REDD1 promotes RAS-mediated tumor progression and metastasis.

### REDD1 loss is sufficient to activate lipid uptake and storage

**Figure 2.**
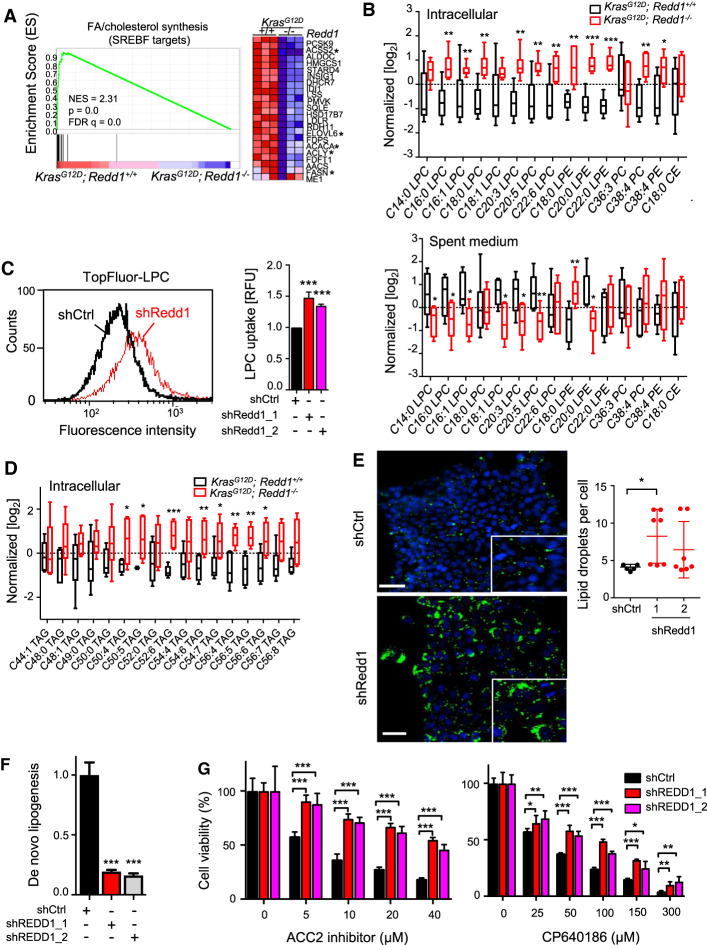
REDD1 deficiency activates lipid uptake and suppresses de novo lipogenesis. (*A*) Gene set enrichment analysis (GSEA) reveals SREBF targets (signature M3009) as the most significantly differentially expressed signature. (*Left*) GSEA plot of RNA-seq data showing suppression of fatty acid (FA) and cholesterol synthesis genes in paired *Kras^G12D^;Redd1*^−/−^ versus *Kras^G12D^; Redd1*^+/+^ primary MEFs (KRMEFs and KMEFs, respectively, cultured under hypoxia (1% O_2_, 18 h). (NES) Normalized enrichment score; (FDR) false discovery rate. (*Right*) Heat map of the entire gene signature shown at *left*. Asterisks indicate FA synthesis genes. (*B*) Levels of phospholipids in KMEFs and KRMEFs (*top*) and their spent medium (*bottom*) collected during growth under hypoxia (1% O_2_) for 18 h as detected by UHPLC-MS lipidomics analysis. Data were normalized by MetaboAnalyst. Vertical lines (whiskers) denote range, and boxes indicate one SD. Triplicate samples from two mice per genotype were analyzed. (LPC) Lysophosphatidylcholine; (LPE) lysophosphatidylethanolamine; (PC) phosphatidylcholine; (PE) phosphatidylethanolamine; (CE) cholesterol ester. (*) *P* < 0.05; (**) *P* < 0.01; (***) *P* < 0.001. (*C*) Histogram showing increased uptake of TopFluor-LPC in REDD1-ablated compared with control KRAS-activated primary pancreatic epithelial cells (KPECs) as assessed by flow cytometry. (*Right*) Quantification of relative fluorescence intensity (RFU) from seven independent experiments is shown as a histogram at the *right*. Error bars indicate SD. (***) *P* < 0.001. (*D*) Unsaturated long chain triglyceride (TAG) levels in KRMEFs compared with KMEFs under hypoxia as detected by lipidomic analysis. Vertical lines (whiskers) indicate range and boxes indicate one SD. Triplicate samples from two mice per genotype were analyzed. (*E*) Lipid droplet (LD) staining via LD540 (green) in shCtrl and shRedd1 KPECs cultured in hypoxia (1% O_2_) for 48 h and visualized by fluorescence microscopy. Nuclei were stained with DAPI (blue). (*Right*) Quantification of LD levels from at least five random fields (dots) of >200 cells/field per genotype. LD numbers were normalized to cell numbers (nuclei) quantitated by DAPI. (*) *P* = 0.013 by two tailed *t*-test. Scale bar, 20 µm. (*F*) De novo lipogenesis, determined from ^14^C-acetate incorporation in A549 cells stably expressing empty vector control (shCtrl) or shRNA (shREDD1). Error bars denote ±SD of three independent experiments. (*G*) Loss of REDD1 decreases reliance on FA synthesis. A549 cells were treated with the specific ACC2 inhibitor (ACC2i) {N-[1-(2′-{4-isopropoxyphenoxy}-2,5′-bithiazol-5-yl)ethyl] acetamide} or the isoform-nonselective ACC inhibitor CP640186 at denoted doses for 72 h. Error bars indicate SD from quintuplicate wells in each of three independent experiments. (*) *P* < 0.05; (**) *P* < 0.01; (***) *P* < 0.001 for all graphs unless noted otherwise. See also Supplemental Figure S2.

As a first step to unravel the physiological basis for REDD1's cooperation with mutant RAS, we developed multiple cell-based models. We found that this cooperation was not restricted to epithelial cells, as *Redd1*^−/−^ MEFs immortalized with adenoviral E1A and expressing ectopic mutant KRAS rapidly formed tumors when injected subcutaneously into immunodeficient mice, while matched KRAS-expressing *Redd1*^+/+^ cells and primary *Redd1*^−/−^ cells without KRAS never formed tumors (Supplemental Fig. S2A). To reveal the relevant gene expression programs associated with REDD1 loss we generated primary *LSL-Kras^G12D/+^*; *Redd1*^−/−^ MEFs (KRMEFs) and matched *LSL-Kras^G12D/+^*; *Redd1*^+/+^MEFs (KMEFs), exposed them to adenoviral Cre (Ad-Cre) and carried out RNA-seq analysis (Fig. 2A; Supplemental Fig. S2B). The most highly significant gene expression differences between KRMEFs and KMEFs were evident under both normoxia and hypoxia, where Gene Set Enrichment Analysis (GSEA) revealed profound suppression of genes regulating cholesterol and fatty acid synthesis (FASyn) as the top differentially expressed signature (Fig. 2A; Supplemental Fig. S2C–E; Engelking et al. 2017). Conversely, we observed increased expression of fatty acid transport genes in KRMEFs compared with KMEFs, including *Fabp3*, *Fabp7*, and *Fabp4*, the latter of which has been implicated in progression and metastasis of ovarian cancer (Supplemental Fig. S2E; Nieman et al. 2011; Bensaad et al. 2014).

To unveil the consequences of these gene expression changes we then carried out analysis of lipid metabolites in KRMEFs and matched KMEFs via ultra-high-performance liquid chromatography-mass spectrometry (UHPLC-MS) under both normoxia and hypoxia (Priolo et al. 2015). A marked intracellular increase in both saturated and unsaturated long and very long chain lipid species was noted in the absence of REDD1, including phosphotidylcholine (PC), phosphoethanolamine (PE), and the lysophospholipidslysophosphotidylcholine (LPC) and lysophosphoethanolamine (LPE) (Fig. 2B; Supplemental Fig. S2F). Concurrent analysis of culture media from these cells demonstrated consistent depletion of the same lipid species from the medium, potentially suggesting cellular uptake (Fig. 2B). Indeed, staining cells with fluorescent-labeled Topfluor-LPC revealed that LPC uptake was significantly increased in *Redd1*^−/−^ MEFs compared with matched wild-type cells, even in the absence of *RAS* mutation (Supplemental Fig. S2G).

Importantly, this phenotype was recapitulated in primary, *RAS* mutant pancreatic epithelial cells. Thus, ablation of REDD1 expression via lentiviral shRNA in KPEC cells followed by exposure to fluorescent-labeled Topfluor-LPC showed a highly significant increase in LPC uptake (Fig. 2C; Supplemental Fig. S2H; Nguyen et al. 2014). Thus, loss of REDD1 is associated with suppression of fatty acid synthesis genes, together with increases in transport genes and uptake of selected lipid species.

Consistent with these findings, increased uptake triggered by REDD1 loss was associated with increased storage of lipids in both epithelial cells and MEFs. We found evidence for increased intracellular synthesis of triglycerides (TAGs), including elevated TAG levels in KRMEFs compared with matched primary KMEFs (Fig. 2D; Supplemental Fig. S2I). As anticipated, these same TAGs were not depleted from the medium (Supplemental Fig. S2J). Lysophospholipids are remodeled and stored together with accumulated TAGs in lipid droplets (LDs), and we therefore tested for the presence of LDs by staining cells with the lipophilic fluorophore LD540 (Spandl et al. 2009). Indeed, we observed increased LD formation in primary KPECs following REDD1 knockdown (Fig. 2E), and in *Redd1*^−/−^ compared with WT MEFs (Supplemental Fig. S2K). These findings suggest that loss of REDD1 is sufficient to induce increased lipid uptake and storage in both the presence and absence of *RAS* mutation.

In order to test whether REDD1 loss induced these changes in human cells we ablated REDD1 via lentiviral shRNA in *KRAS* mutant A549 carcinoma cells. Loss of REDD1 indeed significantly increased LPC uptake (Supplemental Fig. S2L,M). We then measured de novo FASyn (i.e., de novo lipogenesis) directly through analysis of ^14^C-acetate incorporation in these cells. We observed dramatic suppression of de novo FASyn resulting from loss of REDD1 (Fig. 2F). Correspondingly, REDD1-ablated cells were less reliant on FASyn, as loss of REDD1 conveyed significant resistance to inhibitors of acetyl CoA carboxylase (ACC), the first committed step of FASyn (Fig. 2G). Taken together, these results reveal that REDD1 loss activates a program of increased lipid uptake and storage, together with suppressed de novo lipogenesis.

### REDD1 loss in *RAS* mutant cells promotes fatty acid oxidation for energy and redox homeostasis

**Figure 3.**
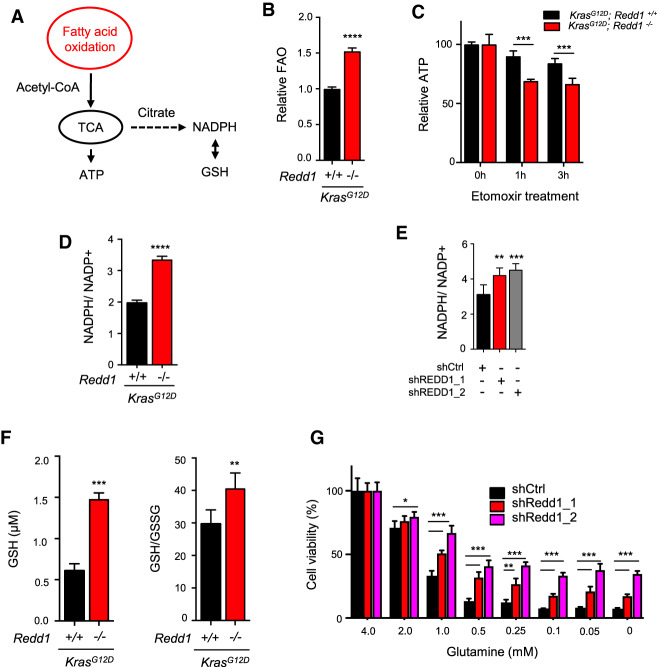
REDD1 loss potentiates fatty acid oxidation (FAO) to drive metabolic dependencies on redox and energy homeostasis. (*A*) Schematic showing proposed pathways linking FAO to redox and energy homeostasis. (*B*) Increased FAO in primary KRMEFs compared with KMEFs under serum starvation for 3 h as measured by ^3^H palmitate labeling. Bar graph shows mean of three experiments. Error bars indicate SD. (*C*) Rapid ATP depletion in KRMEFs compared with KMEFs with FAO inhibition by etomoxir treatment (250 µM) for 1 h, assessed by ATP Cell Titer-Glo assay. Bar graph shows mean from duplicate experiments. (*D*) Increased NADPH/NADP^+^ ratio in KRMEFs compared with KMEFs, assessed as described in the Materials and Methods. Graph shows mean of two experiments performed in duplicate. (*E*) Increased NADPH/NADP^+^ ratio in REDD1-ablated primary KPECS. Bars indicate mean of two experiments performed in duplicate. (*F*) Increased GSH concentration and GSH/GSSG ratio in KRMEFs as compared with KMEFs. (*G*) REDD1 ablation renders KPECs resistant to glutamine withdrawal. Cells were cultured in the denoted concentrations for 3 d. Data represents mean of three independent experiments. See also Supplemental Figure S3. Unless noted otherwise, all error bars denote SD. (*) *P* < 0.05; (**) *P* < 0.01; (***) *P* < 0.001; (****) *P* < 0.0001 by two-tailed *t*-test for all panels.

Under stress conditions including hypoxia, enhanced lipid uptake and storage is linked to catabolism of stored lipids through fatty acid oxidation (FAO), which can contribute to homeostasis through multiple mechanisms including ROS detoxification (via generation of NADPH) and relief of energy stress (via oxidative ATP generation) (Fig. 3A; Bensaad et al. 2014; Patra and Hay 2014; Henne et al. 2018). We measured FAO by tracing the fate of tritiated palmitate in *Kras* mutant KMEFs and KRMEFs, and demonstrated that loss of REDD1 significantly induces FAO in this context (Fig. 3B). These *Redd1*-null cells were highly dependent on FAO for ATP generation, as inhibition of FAO with etomoxir significantly attenuated ATP levels in KRMEFs compared with KMEFs (Fig. 3C). Furthermore, increased FAO in KRMEFs was accompanied by decreased ROS (Supplemental Fig. S3A), and conversely, restoration of REDD1 expression increased ROS levels (Supplemental Fig. S3B). In agreement with these findings, NADPH levels were substantially elevated in KRMEFs compared with KMEFs (Fig. 3D), as they were following REDD1 knockdown in KPECs (Fig. 3E). Notably, REDD1-dependent differences in ROS between KRMEFs and KMEFs were not associated with any change in the antioxidant response factor NRF2, which is increased in a subset of *RAS* mutant tumors (Supplemental Fig. S3C; DeNicola et al. 2011).

We then tested the functional consequences of increased NADPH in this setting, demonstrating that REDD1 knockdown in KPECs was sufficient to confer oxidative stress resistance (Supplemental Fig. S3D). NADPH provides reducing power to generate the key cellular antioxidant reduced glutathione (GSH) and, as expected, both the relative and absolute levels of GSH were increased in the absence of REDD1 (Fig. 3F). Furthermore, we tested the glutamine dependence of these cells, as glutamine has been reported to be required for NADPH generation, ROS detoxification and proliferation in some *RAS* mutant cells (Son et al. 2013; Romero et al. 2017). Consistent with the hypothesis that FAO rather than glutamine meets these metabolic demands in the setting of REDD1 loss, knockdown of REDD1 rendered KPECs substantially less sensitive to glutamine withdrawal than controls (Fig. 3G). Thus, loss of REDD1 in *RAS* mutant cells increases FAO, on which these cells depend for ATP generation and ROS detoxification.

### Increased lipid storage and redox dependence in *RAS* mutant, REDD1-deficient tumors in vivo

**Figure 4.**
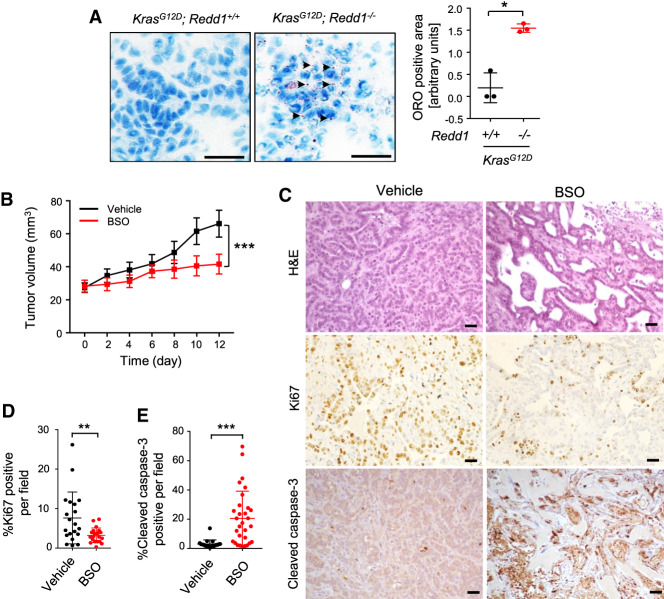
REDD1-deficient tumors show increased lipid storage and glutathione dependence. (*A*) Accumulation of neutral lipid droplets analyzed by Oil Red O (ORO) staining of matched *AdK* and *AdKR* primary lung tumor sections, showing characteristic speckled droplets only in *AdKR* tumors. Scale bars, 20 µm. (*Right*) Summary quantification of ORO staining in lung and pancreatic tumors of the indicated genotypes. Values represent mean ± SEM. (*) *P* = 0.030, by paired *t*-test. (*B*) *AdKR* orthotopic tumors are responsive to glutathione synthase inhibitor buthionine sulfoximine (BSO). Cells from tumors shown in Figure 1E were implanted subcutaneously, and BSO (10 mmol/kg, daily) was administered by i.p. injection 14 d after implantation (=day 0). (***) *P* < 0.001 by one-way ANOVA. (*C*) Representative sections of *AdKR* orthotopic tumors stained by H&E (*top*), Ki67 (*middle*), and cleaved caspase-3 (*bottom*) after 12-d treatment with vehicle or BSO. Scale bars, 20 µm. (*D*) Quantification of Ki67-positive cell percentage from *AdKR* orthotopic mouse tumor sections from *C*. (*E*) Quantification of cleaved caspase-3 positive cells from *AdKR* orthotopic mouse tumor sections from *C*. For *D* and *E*, five random fields per tumor from each of five randomly selected tumors were quantitated. Data represent mean ± SD. (*) *P* < 0.05; (**) *P* < 0.01; (***) *P* < 0.001, by two-tailed *t*-test. See also Supplemental Figure S4.

The key hallmark of rewired lipid metabolism we identified in *RAS* mutant/REDD1-deficient cells is increased lipid uptake, resulting in accumulation of Lipid Droplets (LDs). To establish whether this phenotype was indeed reflected in the in vivo models, we tested lipid storage by staining primary *Kras* mutant lung and pancreas tumors from *Redd1* mutant or wild-type mice with Oil Red O (ORO). While LDs were not identified in the pancreatic or lung tumors of *p48K* or *AdK* mice, respectively, the tumors in *p48KR* and *AdKR* mice demonstrated abundant ORO-positive LDs (Fig. 4A). Thus, loss of REDD1 increases lipid storage in *RAS* mutant tumors in vivo.

Our findings suggest that increased lipid storage and activated FAO in this context promotes tumor progression through enhanced ROS detoxification via NADPH generation and increased reduced glutathione (GSH) (Fig. 3). We therefore sought to assess the requirement for such ROS detoxification in REDD1/RAS-associated tumors in vivo, using an allograft model involving implantation of primary *AdKR* lung carcinomas into immunodeficient mice. We found that this approach produces visibly progressive tumors within 2 wk (Fig. 4B). We assessed dependence on GSH-mediated ROS detoxification in these tumors by blocking glutathione synthesis using buthionine sulfoxamine (BSO) (Harris et al. 2015; Chio et al. 2016). Treatment of tumor-bearing mice with BSO consistently arrested progression of *AdKR* tumors (Fig. 4B; Supplemental Fig. S4). Even more striking was the histologic appearance of posttreatment tumors. Untreated tumors showed uniform fields of viable cells with a high proliferative index as assessed by Ki67 staining. In contrast, BSO-treated tumors showed decreased Ki67 expression and increased apoptosis, evidenced by immunohistochemistry strongly positive for activated Caspase 3 (Fig. 4C–E). Thus, loss of REDD1 increases GSH levels, on which these aggressive tumors are highly dependent for viability in vivo.

### A HIF1α/PPARγ/CD36 axis activated by REDD1 deficiency contributes to lipid uptake and cell migration

**Figure 5.**
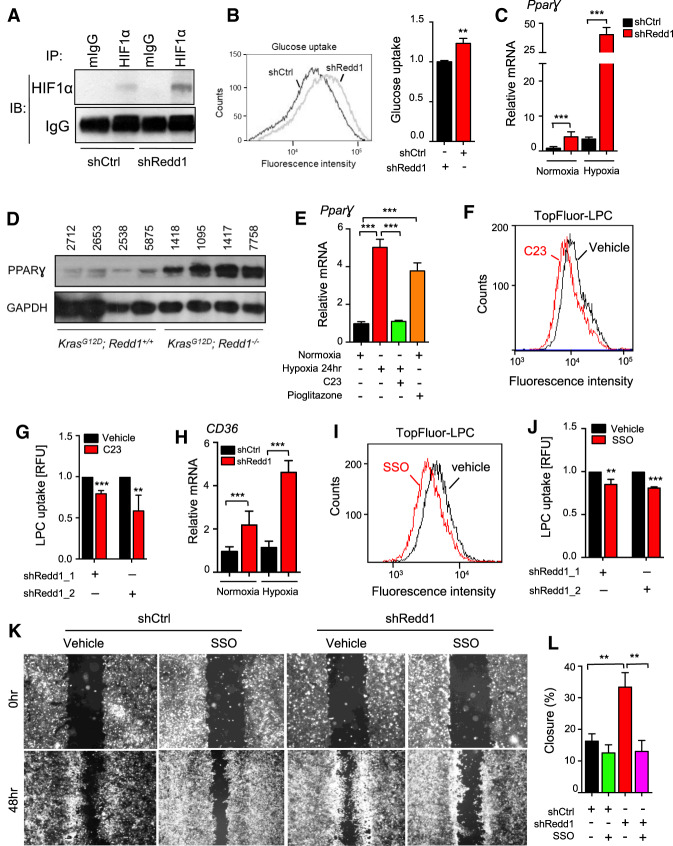
REDD1-dependent HIF1α activation induces the PPARγ-CD36 fatty acid transport pathway and promotes cell migration. (*A*) Knockdown of REDD1 in primary KPECs increases the protein level of HIF1α as detected by IP/Western analysis. IgG serves as a control for IP. (*B*) Histogram showing increased uptake of fluorescent glucose analog 2-NBDG in REDD1-ablated KPECs, assessed by flow cytometry. (*Right*) Summary data from three independent experiments. (*C*) mRNA expression levels of PPARγ in KPECs following shCtrl and shRedd1, as measured by qRT-PCR. Cells were cultured under normoxia or hypoxia (1% O_2_) for 18 h. Graph shows mean of two experiments performed in duplicate. (*D*) Western analysis of primary tumors from *AdK* and *AdKR* mice, demonstrating an increase in PPARγ protein in the absence of REDD1. (*E*) Cotreatment with HIF1α inhibitor C23 (60 µM) blocks induction of PPARγ under hypoxia (1% O_2_) for 24 h in shRedd1 primary KPECs, as measured by qRT-PCR. Pioglitazone serves as a positive control for PPARγ induction. (*F*) C23 treatment (40 µM) for 12 h decreased uptake of Topfluor-LPC in shRedd1 primary KPECs as measured by flow cytometry. (*G*) Summary of three independent experiments performed as in *F*, using two distinct REDD1-directed shRNAs. (*H*) mRNA expression levels of CD36 in shCtrl and shRedd1 primary KPECs, as measured by qRT-PCR. Cells were cultured under normoxia or hypoxia (1% O_2_) for 18 h. Graph shows mean of two experiments performed in duplicate. (*I*) CD36 inhibitor SSO (200 µM) for 12 h blocked uptake of Topfluor-LPC in shRedd1 primary KPECs as measured by flow cytometry. (*J*) Summary of three independent experiments performed as in *I*, using two distinct REDD1-directed shRNAs. (*K*) Representative images showing the scratch (wound) at time 0 and 48 h with and without SSO treatments (200 µM) in shRedd1 KPECs cultured under hypoxia (1% O_2_). Uniform cassette inserts were removed to create 0.9 mm wound at time 0 h. The same area was pictured on time 0 and 48 h. (*L*) Quantification of wound closure. Percent wound closure was measured using Image J. Shown are the means of three independent experiments. Error bars represent SEM. Unless otherwise noted, for all graphs. (**) *P* < 0.01; (***) *P* < 0.001, by two-tailed *t*-test. See also Supplemental Figure S5.

A clue to the mechanism of altered REDD1-dependent lipid metabolism was our observation that multiple genes deregulated in *REDD1/RAS* mutant cells, including fatty acid transport genes (Supplemental Fig. S2), are transcriptional targets of the Hypoxia-Inducible Factor HIF1 (Bensaad et al. 2014). Indeed, *REDD1* is a hypoxia-regulated gene whose loss has been linked to stabilization of HIF1α (Brunelle et al. 2005; Horak et al. 2010). HIF1α levels were in fact increased in primary KPECs following REDD1 knockdown compared with controls, under both normoxia and hypoxia (Fig. 5A; Supplemental Fig. S5A), and in KRMEFs compared with matched KMEFs (Supplemental Fig. S5B). Accordingly, expression of the key HIF1 target gene encoding the glucose transporter GLUT1 was markedly elevated (Supplemental Fig. S5C), and was associated with increased glucose uptake in REDD1-deficient KPECs (Fig. 5B). Further supporting a glycolytic phenotype, oxygen consumption and maximal respiratory capacity were suppressed in *Ras* mutant, REDD1-deficient cells compared with *Ras* mutant controls (Supplemental Fig. S5D). In addition, steady-state metabolite profiling using LC-MS demonstrated accumulation of pyruvate and lactate, with concurrent suppression of pentose phosphate pathway and TCA metabolites in the REDD1-deficient cells (Supplemental Fig. S5E). Thus, HIF1α, its target genes and a glycolytic phenotype are induced due to REDD1 loss.

HIF1α has been linked to fatty acid uptake and lipid storage in cardiomyocytes under pathologic stress through a novel pathway involving activation of the peroxisome proliferator-activated receptor γ (PPARγ) (Krishnan et al. 2009). PPARγ is known to play an important role in lipid metabolism, promoting fatty acid uptake and TAG accumulation in multiple tissues (Spiegelman 1998). We indeed found that the increase in HIF1α observed with loss of REDD1 in both KPECs and MEFs was associated with increased mRNA for PPARγ, which, like HIF1α itself, is further up-regulated under hypoxia (Fig. 5C; Supplemental Fig. S5F). Similarly, PPARγ protein was highly up-regulated in lung tumors of REDD1-deficient *AdKR* compared with *AdK* mice (Fig. 5D). We then tested whether HIF1α was required for REDD1-associated PPARγ induction. Using three distinct HIF1α inhibitors, we found that induction of PPARγ in KPECs following REDD1 knockdown was HIF dependent (Fig. 5E; Supplemental Fig. S5G). Furthermore, we found that lysophospholipid uptake was promoted through this HIF1α/PPARγ pathway, as inhibition of HIF1α or applying a PPARγ antagonist significantly reduced LPC uptake in KPECs following REDD1 knockdown (Fig. 5F,G; Supplemental Fig. S5H,I).

Among the key target genes of PPARγ we found to be induced in the setting of REDD1 deficiency is the *CD36* receptor. We observed increased CD36 expression in KPECs after REDD1 knockdown (Fig. 5H), in KRMEFs compared with KMEFs (Supplemental Fig. S5J), and in vivo in human pancreas carcinomas with low REDD1 levels (Supplemental Fig. S5K). CD36 is at the apex of a signaling cascade involved in lipid uptake and storage, through which it has recently been shown to enable tumor metastasis (Pascual et al. 2017). We implicated CD36 in lipid transport in these cells, as treatment with the irreversible CD36 inhibitor Sulfosuccinimidyl Oleate (SSO) substantially blocked LPC uptake (Fig. 5I,J; Coort et al. 2002). CD36 engenders metastasis by promoting a sub-population of slow-cycling cells with enhanced metastatic capacity (Pascual et al. 2017). To explore such phenotypes in the REDD1 context we used an in vitro wound healing assay, in which migration across a uniform gap is quantitated (Kramer et al. 2013). REDD1 knockdown in KPECs consistently enhanced migration across the gap, and this effect was abolished by inhibition of CD36 with SSO (Fig. 5K,L). Taken together, these observations imply that *RAS* mutant, REDD1-deficient cells co-opt a HIF1α/PPARγ-dependent pathway for pathological lipid storage characteristic of cardiomyocytes, while activating CD36 to promote lipid uptake, altered metabolism and enhanced cell migration.

### Decreased REDD1 levels are associated with poor outcomes selectively in *RAS* mutant human cancers

**Figure 6.**
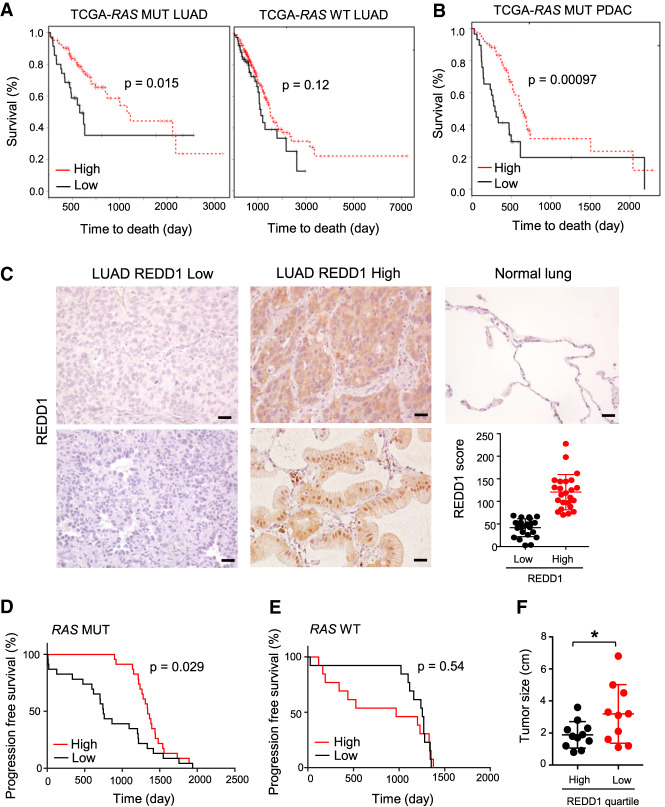
Low REDD1 levels confer worse outcomes in *RAS* mutant human cancers. (*A*) Kaplan-Meier analysis of patient outcomes in the TCGA lung adenocarcinoma (LUAD) cohort, applying the REDD1 gene expression signature (see the text). (*Left*) Shorter survival is seen among *RAS* mutant LUAD patients with low REDD1 signature. Comparison of bottom (*n* = 35, median survival 656 d) and top (*n* = 104, median survival 1653 d) quartiles of metagene values. (*Right*) No significant difference in survival among *RAS* WT patients based on the REDD1 signature. Comparison of bottom (*n* = 77, median survival 1115 d) and top (*n* = 230, median survival 1499 d) quartiles of metagene values. *P*-values calculated by log-rank test. (*B*) Kaplan-Meier analysis of patient outcomes in the TCGA pancreatic adenocarcinoma (PDAC) cohort, applying the REDD1 signature. Shorter survival is observed among *RAS* mutant PDAC patients with low REDD1 signature. Comparison of bottom (*n* = 29, median survival 293 d) and top (*n* = 87, median survival 634 d) quartiles of metagene values. (*C*) IHC for REDD1 using affinity-purified anti-REDD1 antibody on lung adenocarcinomas (LUAD; *n* = 47 cases) and normal lung tissue. Representative photomicrographs of REDD1 low (*left*) and high (*middle*) LUAD cases. Scale bars, 20 µm. (*Bottom*, *right* panel) Distribution of REDD1 IHC scores among 47 cases. (*D*) Kaplan-Meier analysis of clinical outcomes based on tumor cell REDD1 protein expression, showing significantly shorter progression free survival (PFS) for patients with *RAS* mutant adenocarcinomas in the bottom 50% of REDD1 IHC scores (median PFS: 749 d) compared with the top 50% of REDD1 IHC scores (median PFS: 1338 d). *P*-values calculated by log-rank test. (*E*) Among *RAS* WT patients, no statistically significant difference in PFS is observed based on REDD1 protein expression. *P*-value by log-rank test. (*F*) Lower REDD1 protein expression is associated with larger primary tumors. Shown are tumors in the *top* and *bottom* quartiles of REDD1 expression. (*) *P* = 0.045 by two tailed *t*-test.

Finally, we tested the hypothesis that *RAS* mutant human tumors with loss of REDD1 expression, like their murine counterparts, may behave particularly aggressively and therefore be associated with especially poor patient outcomes. Thus, we developed a gene expression signature of REDD1-loss from matched *REDD1/RAS* versus *RAS* mutant cells (see Materials and Methods), then we determined the ability of this signature to predict patient outcomes in a large clinical data set (TCGA) (The Cancer Genome Atlas Research Network 2014, 2017). Using a signature of REDD1 loss was required, as tumor cell levels of REDD1 itself in bulk RNA-seq data are confounded by substantially higher expression in nonepithelial than epithelial cells (Supplemental Fig. S6A). We observed that the median survival of patients with *RAS* mutant tumors expressing the lowest levels of the REDD1 signature (that is, associated with REDD1 loss) was approximately one-third that of those whose tumors expressed the highest levels of the signature (*P* = 0.015, Fig. 6A). Remarkably, no statistically significant association with survival was observed based on the REDD1 signature among those with *RAS* wild-type tumors (*P* = 0.12) (Fig. 6A). Also of note, the association between the REDD1 signature and outcomes in *RAS* mutant tumors was not confounded by smoking status (Supplemental Fig. S6B) or by additional prognostic somatic mutations, as there was no statistically significant association between the REDD1 signature and the presence/absence of such mutations, including *TP53* and *KEAP1* in this cohort. We then analyzed the TCGA cohort for PDAC, the large majority of which harbor *RAS* mutations (The Cancer Genome Atlas Research Network 2014, 2017). Again, the REDD1 loss gene expression phenotype predicted worse outcome with high statistical significance (*P* = 0.00097), even considering the overall dismal survival of PDAC as a whole (Fig. 6B).

**Figure 7.**
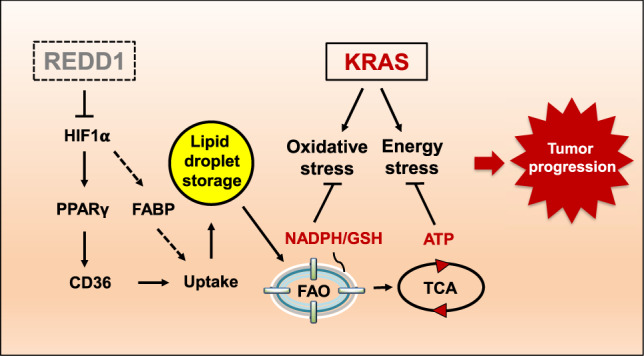
Model for REDD1 loss in RAS-driven tumorigenesis. REDD1 loss up-regulates HIF, PPARγ/CD36, and fatty acid-binding proteins (FABPs) to mediate uptake and lipid droplet storage. This effect is associated with increased fatty acid oxidation (FAO) that generates ATP as well as reducing equivalents (NADPH and GSH) through the mitochondrial Krebs (TCA) cycle, resulting in relief from RAS-induced energy stress and oxidative stress, and ultimately tumor progression. We hypothesize that combination therapies targeting the multiple metabolic nodes described herein are likely to be effective against REDD1-deficient, *RAS* mutant cancers.

We further credentialed these findings by direct analysis of REDD1 protein in tumors via immunohistochemistry. We developed and validated a high specificity, affinity-purified anti-REDD1 antibody (DeYoung et al. 2008) that we applied to a clinically annotated set of primary lung adenocarcinomas (Huynh et al. 2016). REDD1 expression is moderate in normal pulmonary epithelia, and we developed a scoring system to identify subsets of *RAS* mutant tumors with both high and low/absent tumor cell expression of REDD1 (Fig. 6C). Despite the relatively small numbers, patients with *RAS* mutant tumors exhibiting low REDD1 protein expression experienced significantly worse progression-free survival (*P* = 0.029, Fig. 6D). As in the TCGA cohort, low REDD1 expression was not associated with worse progression-free survival among those whose lung tumors lacked *RAS* mutation (Fig. 6E). Furthermore, *RAS* mutant tumors with low REDD1 protein levels were found to be significantly larger at time of diagnosis that REDD1-high tumors, consistent with a role for REDD1 loss in particularly aggressive disease (Fig. 6F). All together, these findings reveal REDD1 loss as a hallmark and driver of a *RAS* mutant tumor subset characterized by a reprogramming of lipid metabolism, rapid progression and poor outcomes (Fig. 7).

## Discussion

Here we reveal that loss of the stress response gene, REDD1, underlies a particularly lethal subgroup of *RAS* mutant tumors. Our studies show that REDD1 deficiency engenders markedly elevated lipid uptake and storage, coupled to a glycolytic phenotype and activated FAO that sustains metabolic demands of the *RAS* mutant context to promote tumor progression including metastatic dissemination. Notably, the contribution of REDD1 to human cancer has to date been unclear, as deletion of REDD1 alone is insufficient to confer tumor predisposition in mice (Lipina and Hundal 2016). However, we found that in the context of activated RAS, loss of REDD1 and the ensuing metabolic rewiring convert preneoplastic lesions into invasive and in some cases metastatic malignancies. A highly relevant functional interaction between REDD1 and mutant RAS is further supported by our observation that REDD1 expression is associated with clinical outcomes selectively in human *RAS* mutant, but not *RAS* wild-type lung and pancreas tumors. Collectively, these findings suggest the REDD1-mediated stress response as a novel tumor suppressor mechanism in the context of *RAS* mutation.

Multiple aspects of lipid metabolism are deregulated in the setting of *RAS* mutation, yet the details of how this rewiring occurs and contributes to cancer pathogenesis remains to be fully elucidated (Biancur and Kimmelman 2018). For example, increased lipid uptake has been described in *KRAS* mutant pancreatic epithelial cells, but the upstream pathways and in vivo consequences were not established (Kamphorst et al. 2013). Furthermore, reports have varied regarding the role of fatty acid synthesis (FASyn) in the context of *RAS* mutation, with some studies suggesting FASyn as an essential contributor and potential therapeutic target in the setting of *RAS* mutation (Singh et al. 2018), while others report reliance instead on uptake and oxidation of fatty acids (Padanad et al. 2016). Here, we show that *RAS* mutant cells and tumors with REDD1 loss exhibit increased lipid uptake, storage, and fatty acid oxidation (FAO), and accordingly that decreased reliance on FASyn is triggered by REDD1 loss even in established human tumor cells (Fig. 2). Taken together, these findings speak to the potential for intertumor heterogeneity of lipid metabolism in this context, and they suggest REDD1 loss as a metabolic switch that activates a unique pathway for lipid deregulation.

Our findings point to activation of HIF1α as a key contributor to the metabolic effects associated with REDD1 loss. The elevated HIF1α levels we observed are consistent with previous work demonstrating that stabilization of HIF1α results from the increased mitochondrial (but not cytosolic) ROS that results from loss of REDD1 (Brunelle et al. 2005; Horak et al. 2010). Furthermore, our results are also in keeping with prior work demonstrating a functional interaction between REDD1 and the stress-induced metabolic regulator TXNIP (Qiao et al. 2015). Like REDD1, loss of TXNIP is associated with stabilization and activation of HIF1α in multiple cellular contexts (Shin et al. 2008; Ji Cho et al. 2019). We found that the activation of HIF1α and PPARγ/CD36 that result from loss of REDD1 are associated with both increased glycolysis and fatty acid/lysophospholipid uptake. Similar effects of HIF1α/PPARγ/CD36 are known to occur under pathologic stress in cardiomyocytes, leading to increased TAG generation from fatty acid conjugation to the glucose-derived glycerol-3-phosphate backbone (Krishnan et al. 2009). While in the latter context pathologic cardiac steatosis results, we provide evidence that HIF1α-dependent lipid storage and an associated increase in FAO confer a distinct advantage to *RAS* mutant tumors. This finding is in keeping with a prior report describing lipid droplet formation and associated increased FAO as a HIF1α-mediated survival mechanism selectively in epithelial tumor cells (Bensaad et al. 2014). Thus, not only is HIF1α/PPARγ/CD36 activation in *RAS/REDD1* mutant tumors associated with dramatic accumulation of lipid storage droplets in vivo, this storage is coupled to increased FAO that we found is important for generation of both ATP and ROS-detoxifying NADPH. We further link FAO and redox homeostasis to REDD1-dependent tumor progression in vivo, demonstrating the critical sensitivity of *RAS/REDD1* mutant tumors to NADPH/GSH-mediated detoxification. Thus, treatment with the glutathione synthesis inhibitor BSO induces growth arrest and cell death within these tumors in vivo.

A consistent feature of the *RAS* mutant, REDD1-deficient phenotype is the progression to invasive malignancy including in some cases disseminated hematogenous metastasis. Our data suggest that this effect relates in part to the up-regulation of the CD36 receptor in this context. CD36 has now been implicated as a mediator of poor outcomes and metastasis in multiple different cancers (Pascual et al. 2017; Ladanyi et al. 2018). Our findings that CD36 is required for lipid uptake and cell migration in REDD1-deficient cells provide new insight into CD36 activation in cancer, and they collectively suggest a direct contribution of CD36 to the pathogenesis of REDD1-deficient tumors. Collectively, these results point to a potential role for lipid metabolism as a driver of invasive and ultimately metastatic disease.

All the points noted above imply that the metabolic rewiring that characterizes *RAS* mutant, REDD1-deficient tumors may be of direct clinical relevance. For example, multiple studies have suggested a particular reliance of certain *KRAS* mutant tumors on glutamine, and glutaminase inhibitors that aim to exploit this dependence have now entered clinical trials (Son et al. 2013; Fung and Chan 2017; Romero et al. 2017). However, the relative insensitivity to glutamine withdrawal we demonstrate in the setting of REDD1 loss may predict that patients presenting with such tumors will be poor candidates for this therapy. Similarly, recent work has documented dependence of *Kras* mutant GEMMs on fatty acid synthesis, resulting in tumor sensitivity to inhibition of the key committed step in this process catalyzed by ACC (Svensson et al. 2016). Again, the REDD1 phenotype is distinct, as REDD1 loss is associated with suppressed fatty acid synthesis in the mutant RAS context, and we show directly that this effect confers substantial resistance to ACC inhibitors. Conversely, we found that REDD1-deficient tumors are highly sensitive to glutathione depletion with BSO. Clinical trials using this agent have not yielded responses in a large proportion of tumors, yet our findings suggest the possibility that targeting a metabolically defined tumor subset, such as that discovered here, may be more successful (Kirkpatrick and Powis 2017). Finally, CD36 itself is an attractive potential therapeutic target whose inhibition has proven relatively nontoxic in preclinical models (Pascual et al. 2017).

In summary, we show that REDD1 deficiency defines and drives progression and poor clinical outcomes in *RAS* mutant tumors. We reveal distinct metabolic rewiring engendered by REDD1 that alleviates RAS-induced metabolic stress and is associated with the progression to invasive disease and metastasis. Collectively, these findings advance our understanding of the heterogeneity of common *RAS* mutant tumors, and they reveal loss of REDD1 as a clinically important and potentially therapeutically actionable hallmark in these malignancies.

## Materials and methods

### GEMMs

Mice were housed in pathogen-free animal facilities at Massachusetts General Hospital. All experiments were performed under protocol 2004N000228, approved by the Subcommittee on Research Animal Care at Massachusetts General Hospital. Mice were maintained on a C57BL/6 background. Data presented include both male and female mice. All mice included in the survival analysis were killed when criteria for disease burden (including abdominal distension that impeded movement, loss of >15% of body weight, labored breathing, and/or abnormal posture) were reached. *Redd1*^−/−^ strain were crossed with *p48-Cre* strain and the *LSL-Kras^G12D^* strain (Jackson Laboratory, from the Bardeesy laboratory), which consisted of a mutant *Kras*^*G12D*^ allele knocked into the endogenous *Kras* locus, preceded by an LSL cassette.

### Adenovirus-induced lung adenocarcinoma

*LSL-Kras^G12D^* (Kras) and *LSL-Kras^G12D^*; *Redd1*^−/−^ mice were treated with 5 × 10^5^ or 5 × 10^6^ plaque-forming units of adenovirus expressing Cre (University of Iowa Adenovirus Core) by intratracheal infection as described previously (DuPage et al. 2009). Tumors were analyzed 12 wk after infection.

### Allograft lung cancer mouse model

*Kras*^*G12D*^; *Redd1*^−/−^ primary tumors were dissociated by collagenase digestion containing soybean trypsin inhibitor, digested samples were teased through a 100-mm filter, resuspended in culture medium. CD31 and CD45 positive cells were discarded through flow cytometry sorting. Tumor cells were then mixed with matrigel and were injected subcutaneously into nude mice in double-flanks. Two weeks later, when tumors reached an average diameter of 4–5 mm, tumor-bearing mice were randomized into two groups with that day as day 0. For in vivo buthionine sulfoximine (BSO; Sigma) treatment, tumor-bearing mice were treated daily with intraperitoneal injections of vehicle or BSO at a dose of 10 mmol/kg dissolved in PBS. Tumors were measured every other day for up to 12 d, and tumor volumes were calculated with the formula: tumor volume (mm3) = 4/3π × length/2 × width/2.

### Orthotopic pancreatic cancer mouse model

SCID mice (C3SnSmn.CB17-Prkdc^scid^/J,; Jackson Laboratories) were subjected to general anesthesia according to Massachusetts General Hospital Subcommittee on Research Animal Care policies. Orthotopic injections of the pancreas were conducted as previously described (Corcoran et al. 2011) using 2 × 10^4^ *p48-Cre; LSL-Kras^G12D^* pancreatic ductal cells suspended in 50 mL of duct medium mixed (Agbunag et al. 2006) with 50 mL of Matrigel (BD Biosciences).

### Primary pancreatic cancer epithelial cells

Primary pancreatic epithelial cells were derived from 9-wk-old *p48-Cre; LSL-Kras^G12D^* mice as described previously (Agbunag et al. 2006). These *Kras*^*G12D*^-acitivated pancreatic epithelial cells were propagated on surfaces coated with laminin (BD Biosciences) and in a specific pancreatic medium.

### Primary mouse embryonic fibroblasts

Early passage MEFs (*P* < 5) were used for all of the experiments. *LSL-Kras^G12D^* mice were crossed with *Redd1*^−/−^ mice to generate *LSL-Kras^G12D^; Redd1*^−/−^ MEFs. Mice and MEFs were on C57BL/6 background. Embryos were genotyped according to published procedures. MEFs were grown in DMEM/10% FCS/Pen/Strep. To activate *Kras* in the *LSL-Kras^G12D^*, cultures were infected with Ad-Cre (500-1000 pfu/cell) leading to activation of *Kras*^*G12D*^ in AdK and AdKR cells.

### Cell culture

The *REDD1*^−/−^ allele was generated as previously described (Sofer et al. 2005). MEFs not harboring *LSL-Kras^G12D^* were immortalized by retroviral transduction of SV40 large T-antigen unless indicated otherwise, and were maintained in DMEM/10% FCS/Pen/Strep. Human non-small cell lung cancer cell line A549 was obtained from the American Type Culture Collection and maintained in DMEM/10% FCS/pen/strep. Primary pancreatic intraepithelial cells (AH375) were isolated from *LSL-KRAS*^*G12D*^ mice as described previously (Corcoran et al. 2011). All pancreatic ductal epithelial cells were routinely maintained in ductal media on laminin-coated plates. Negative mycoplasma contamination status of all cancer cell lines, 293T cell line and primary cells used in the study was established using LookOut mycoplasma PCR kit (Sigma MP0035).

### Metabolite profiling

#### IC method

A Thermo Scientific Dionex ICS-5000^+^ HPIC ion chromatography system consisting of a dual pump, an eluent generator (EG) with a capillary KOH cartridge, and a detection compartment (DC) featuring an IC module with suppressed conductivity detection was used in this study. The whole system was metal free. The ERS 500 suppressor was operated in external-water mode with ultrapure Milli-Q water, and regenerant was delivered by pressure using an argon gas tank at a pressure of 20 psi. The eluent of the IC system was converted to pure water after the column and flowed directly to the MS source. To help with the desolvation for better electrospray, makeup solvent of methanol was delivered at 60 μL/min and mixed with the eluent via a low dead volume mixing tee, and passed through a grounding union before entering the MS. The HPIC analysis was conducted with the IonPac AS11HC-4 μm, 2.0 × 250-mm columns (2000 Å). IC flow rate was 380 μL/min (at 30°C supplemented postcolumn with makeup flow). The gradient conditions were as follows: An initial 25 mM KOH was held for 3 min prior to injection, increased linearly to 95 mM at 8 min, held at 95 mM for 1 min, followed by a drop to 25 mM within 0.1 min, and held for 10 min to re-equilibrate the column. The total run time was 23 min. The ion spray voltage was −3 kV and the source temperature was 350°C. Raw data were taken using full scan analysis over *m/z* 67–1000 at 140,000 resolution. LC-MS data were processed and visually checked using TraceFinder 3.3 software (Thermo Fisher Scientific). Medium samples (30 μL) were derived using 120 μL of 80% methanol (VWR) containing the internal standards inosine-^15^N_4_, thymine-d_4_, and glycocholate-d_4_ (Cambridge Isotope Laboratories). The samples were centrifuged at 9000*g* for 10 min at 4°C, and 5 µL of supernatant was transferred to an autosampler vial and injected on to the HPIC. Cellular extracts were put directly into the system.

#### C8-pos and HILIC-pos methods

Lipid extracts were analyzed using a Nexera X2 U-HPLC (Shimadzu) coupled to an Exactive Plus orbitrap mass spectrometer (Thermo Fisher Scientific). Extracts (10 μL) were injected onto an Acquity UPLC BEH C8 column (1.7 μm, 2.1 × 100 mm; Waters). The column was initially eluted isocratically at a flow rate of 450 μL/min with 80% mobile phase A (95:5:0.1 [v/v/v] 10 mM ammonium acetate/methanol/formic acid) for 1 min, followed by a linear gradient to 80% mobile-phase B (99.9:0.1 [v/v] methanol/formic acid) over 2 min, a linear gradient to 100% mobile phase B over 7 min, and then 3 min at 100% mobile phase B. MS analyses were performed using electrospray ionization in the positive ion mode (source voltage was 3 kV, source temperature was 300°C, sheath gas was 50.0, auxillary gas was 15) using full scan analysis over *m/z* 200–1100 and at 70,000 resolution. Hydrophilic interaction liquid chromatography (HILIC) analyses of water soluble metabolites were carried out in the positive ion mode using a Nexera X2 U-HPLC (Shimadzu)-Q Exactive Orbitrap (Thermo Fisher Scientific) LC-MS instrument. Cell extracts (10 μL) were diluted using 90 μL of 74.9:24.9:0.2 (v/v/v) acetonitrile/methanol/formic acid containing stable isotope-labeled internal standards (valine-d8 [Isotec] and phenylalanine-d8 [Cambridge Isotope Laboratories]). The samples were centrifuged at 9000*g* for 10 min at 4°C and the supernatants were injected directly onto a 150 × 2-mm Atlantis HILIC column (Waters). The column was eluted isocratically at a flow rate of 250 μL/min with 5% mobile phase A (10 mM ammonium formate, 0.1% formic acid in water) for 1 min followed by a linear gradient to 40% mobile phase B (acetonitrile with 0.1% formic acid) over 10 min. The electrospray ionization voltage was 3.5 kV and data were taken using full scan analysis over *m/z* 70–800 at 70,000 resolution. HILIC analyses of water-soluble metabolites in the negative-ion mode were performed using a Nexera X2 U-HPLC (Shimadzu) coupled to a Q-Exactive Plus Orbitrap mass spectrometer (Thermo Fisher Scientific). Cell extracts (10 μL) were injected onto a 150 × 2.0-mm Luna NH2 column (Phenomenex) that was eluted at a flow rate of 400 μL/min with initial conditions of 10% mobile phase A (20 mM ammonium acetate, 20 mM ammonium hydroxide in water) and 90% mobile phase B (10 mM ammonium hydroxide in 75:25 [v/v] acetonitrile/methanol) followed by a 10-min linear gradient to 100% mobile phase A. MS full-scan data were acquired over *m/z* 70–750. Instrument settings were source voltage −3.0 kV, source temperature 325°C, capillary temperature 350°C, sheath gas 55, auxiliary gas 10, and resolution 70,000. LC-MS data were processed and visually inspected using TraceFinder 3.1 software (Thermo Fisher Scientific) LC-MS (Mascanfroni et al. 2015).

### Retroviral and lentiviral production

Viruses expressing pLPC-REDD1-HA, Redd1, and REDD1 were used in this study. shRedd1 clones were cotransfected into HEK293T cells with lentiviral packaging plasmids by using CalPhos mammalian transfection kit (Clontech Laboratories) according to manufacturer's instruction. The conditioned medium containing lentiviral particles was collected 36 h after transfection and filtered with a 0.45-μm pore filter (Milipore). The filtered media was then used to infect target cells. Polybrene (Sigma) was supplemented at final concentration of 10 μg/mL to increase infection efficiency. The infected cells were selected using 1 μg/μL puromycin (Sigma) 72 h after infection. Genetic knockdown of Redd1 was confirmed via either immunoblotting or qRT-PCR analysis. shRNA sequences for Redd1 were GCTATCTTACAGACGCATGAA and GTGTAGCATGTACCTTATTAT. shRNA sequences for REDD1 were GCTATCTTACAGACGCATGAA and GCTATCTTACAGACGCATGAA.

### Immunohistochemistry

Mouse tissue specimens were fixed in 4% buffered formalin for 24 h and kept in 70% ethanol until paraffin embedding. Assistance in processing of murine tumor samples was offered by the Dana-Farber/Harvard Cancer Center Specialized Histopathology Core Facility. Five-micron sections were taken from formalin-fixed, paraffin-embedded tumors and stained using standard protocols. Lung cancer patient tissue microarray were generated at Massachusetts General Hospital Pathology department. Staining for cleaved caspase 3 was performed by Massachusetts General Hospital Histopathology Research Core. Staining for human REDD1 using affinity-purified antibody were conducted by the core staff. IHC results were further evaluated by H-score system or (“histo” score) to tumor samples. The H-score included the sum of individual H-scores for each intensity level seen (score 0: no positive staining; score 3: strongest staining) using the following formula: [0 × (% cells 0) + 1 × (% cells 1+) + 2 × (% cells 2+) + 3 × (% cells 3+). Thus, there were 300 possible values. Using this system, <1% positive cells was considered to be a negative result.

### Staining of LD with LD540 or Oil Red O

For microscopic analysis of LD540 staining, cells were fixed with 4% paraformaldehyde (PFA) for 15 min, incubated with a solution of 0.05 mg/mL LD540 (stock solution in ethanol at 0.5 mg/mL) in PBS in the dark for 10 min at room temperature , and washed three times with PBS. Cells were stained in 1 µg/mL DAPI (Sigma) and mounted in VectaShield mounting medium (Vector Laboratories H-1000). Images were captured with the Olympus IX81 inverted fluorescence microscope. The quantification was performed by counting at least five random fields of view with a 60× objective, and calculating the average and standard deviation. For Oil Red O staining, murine tumors were snap-frozen in HistoPrep (Fisher Scientific), and 5-μm sections were stained with the Abcam Oil Red O (lipid stain) kit (ab150678) according to the manufacturer's instructions. ImageJ was used to quantitate LD levels from microscopic images.

### Flow cytometry

To examine glucose uptake, 100 mM of the fluorescent glucose analog 2-NBDG (Invitrogen) was added to cell culture medium 16 h before analysis. For ROS measurement, cells were stained in full medium at final concentrations of 5 μM CM-H2DCFDA (Sigma) for 40 min in the dark at 37°C. Cells were trypsinized and single-cell suspensions were analyzed by flow cytometry. To measure in vitro transport of LPC, TopFluor LPC was dissolved in 12% BSA, which was diluted in 150 mM NaCl. Uptake of TopFluor LPC in cells were then stained for 20 min at 37°C and cells were then harvested for measurement of flow cytometry analysis.

### Seahorse XFe96

The cellular oxygen consumption rate of live cells was assessed using the Seahorse XFe96 (Seahorse Biosciences). Respiration was measured under basal conditions as well as in the presence of mitochondrial inhibitor 0.25 µM oligomycin, 5 µM uncoupler carbonyl cyanide-4-(tri-fluoromethoxy)phenylhydrazone (FCCP), and 1 µM respiratory chain inhibitors antimycin A and rotenone. Results were normalized to protein concentration. ATP content of cells was determined using the Cell Titer-Glo kit (Promega). Luminescence was normalized to number of cells as measured by DNA content using CyQuant NF.

### Cell viability and proliferation

For growth assays, cells were plated at a density of 1500 cells per well in black 96-well plates using five to six replicates per time point and/or condition, and allowed to attach overnight. Cell viability was measured by ATP content measurement using the CellTiter-Glo luminescent assay (Promega) according to the manufacturer's instructions. Cell growth was assessed by DNA content measurement using the CyQuant NF kit according to the manufacturer's instructions. Day 0 was considered the day after the initial seeding.

### Fatty acid oxidation and lipogenesis

For fatty acid oxidation, primary MEFs were serum starved for 3 h and incubated overnight in culture medium containing 100 mM palmitate (C16:0) and 1 mM carnitine. In the final 2 h of incubation, cells were pulsed with 1.7 µM Ci[9,10(n)-3H] palmitic acid (GE Healthcare), and the medium was collected and eluted on ion exchange columns packed with DOWEX 1X2-400 resin (Sigma) to analyze the released ^3^H_2_O, formed during cellular oxidation of [3H] palmitate. Primary MEFs used for this assay were below passage 5.

For the measurement of lipogenesis, A549 cells were cultured, placed overnight in low-glucose low-serum medium, then labeled with 1-^14^C acetic acid (Perkin Elmer) while stimulated with serum-containing medium for 1 h. Cells were washed twice with PBS before lysis in 0.5% Triton X-100. The lipid fraction was isolated by the addition of chloroform and methanol (2:1 [v/v]), followed by the addition of water. Samples were centrifuged, and ^14^C incorporation was measured in the bottom, lipid-containing phase using a scintillation counter. Each condition was normalized to protein concentrations.

### NADPH, NADH, and GSH assays

Cells were seeded at 5000 cells/well in a 96-well plate. Assays were performed according to instructions from Promega (NADP/NADPH-Glo [G9081], NAD/NADH-Glo [G9071], and GSH/GSSG-Glo assay [V6611]).

### PCR

Total RNA extraction, cDNA synthesis, and qRT-PCR were carried out as described previously. The expression of each gene was normalized to b-actin. Primers used were as follows: Redd1 (5′-GACAGCAGCAACAGTGGCTTC-3′ and 5′-CCACGCTATGGCAGCTCTTGC-3′), Actb (5′-CCAACCGTGAAAAGATGACC-3′ and 5′-CCAGAGGCATACAGGGACAG-3′), PPARγ (5′-CCACCAACTTCGGAATCAGCT-3′ and 5′-TTTGTGGATCCGGCAGTTAAGA-3′), and CD36 (5′-TCCTCTGACATTTGCAGGTCTATC-3′ and 5′-AAAGGCATTGGCTGGAAGAA-3′).

### Co-IP and Western blot

Western analysis was conducted as described previously (Qiao et al. 2015). For immunoprecipitation (IP)/Western analysis of HIF-1α, cells were lysed in RIPA buffer, and equal amounts of total lysate (0.75–1.5 mg) were incubated with anti-HIF1α antibody for 90 min or overnight at 4°C. HIF1α protein was precipitated using Protein G Sepharose (GE Healthcare) for 30 min at 4°C, washed three times with lysis buffer, and visualized by SDS-PAGE. For Western blot of Nrf2 and HIF-1α, nuclear proteins were isolated using NE-PER nuclear and cytoplasmic extraction reagents (Thermo Fisher 78833). Blots were incubated with antibodies recognizing the following proteins: anti-HIF-1α (Novus Biologicals NB100-105), anti-lamin-B1 (Abcam ab16048), Nrf2 rabbit monoclonal antibody (Dr. Edward E. Schmidt, Montana State University), REDD1 (Bethyl Laboratories A302-169A), b-tubulin (Millipore MAB 3408), Ras (G12D mutant-specific, rabbit mAb; Cell Signaling 14429),

### RNA-seq and bioinformatics analysis

RNA sequencing was performed using total RNA isolated from at least three independent mouse embryos per genotype. RNA-seq library preparation and sequencing were performed by Massachusetts General Hospital NextGen Sequencing Core. Gene set enrichment analysis (GSEA) analysis of RNA-seq data was used to generate an unselected statistical ranking of differentially expressed gene signatures. The meta-gene signature applied to TCGA LUAD and PDAC cohorts was derived from RNA-seq results of KRMEFs compared with KMEFs (*n* = 4 samples per genotype). Following normalization and variation filtering, a *t*-test was performed to find genes significantly varying between genotypes with a *P*-value <0.05 and corrected for multiple hypothesis testing. Ultimately, 339 statistically differentially expressed genes could be mapped to TCGA RNA-seqV2 and were used to generate a metagene value used for the analysis (see the Supplemental Material for details).

### Wound healing assay

Experiments were performed according to instructions provided by CytoSelect 24-well wound healing assay (Cell Biolabs CBA-120).

### Statistical analysis

For qRT-PCR analysis, proliferation assays, glucose uptake, tumor sphere formation, and tumor size, significance was analyzed using two-tailed Student's *t*-test. A *P*-value of <0.05 was considered statistically significant. A log-rank test was used to determine significance for Kaplan-Meier analyses. *P*-values are depicted in the figures as follows: *P*-value < 0.05 (*), *P*-value < 0.01 (**), *P*-value < 0.001 (***), *P*-value < 0.0001 (****), and not significant (ns).

## Supplementary Material

Supplemental Material
