# Supplementary material for: REDD1 loss reprograms lipid metabolism to drive progression of RAS mutant tumors
Source: Genes Dev. 2020 Jun 1;34(11-12):751–66. doi: 10.1101/gad.335166.119 (PMC7263146; doi:10.1101/gad.335166.119)
Supplement: Supplemental Material [file supp_34_11-12_751__index.html]

REDD1 loss reprograms lipid metabolism to drive progression of RAS mutant tumors — Supplemental Material 

# REDD1 loss reprograms lipid metabolism to drive progression of *RAS* mutant tumors

## Supplemental Material

- Supplemental\_Data.pdf
