# Supplementary material for: REDD1 loss reprograms lipid metabolism to drive progression of RAS mutant tumors
Source: Genes Dev. 2020 Jun 1;34(11-12):751–66. doi: 10.1101/gad.335166.119 (PMC7263146; doi:10.1101/gad.335166.119)
Supplement: Supplemental Material [file supp_gad.335166.119_Supplemental_Data.pdf]

Qiao_Supplemental Materials

Supplemental Information Table of Contents

Supplemental Figure 1, related to Figure 1

Supplemental Figure 2, related to Figure 2

Supplemental Figure 3, related to Figure 3

Supplemental Figure 4, related to Figure 4

Supplemental Figure 5, related to Figure 5

Supplemental Figure 6, related to Figure 6

Supplemental Methods

Supplemental References

Qiao_Supplemental Figure 1, related to Figure 1.

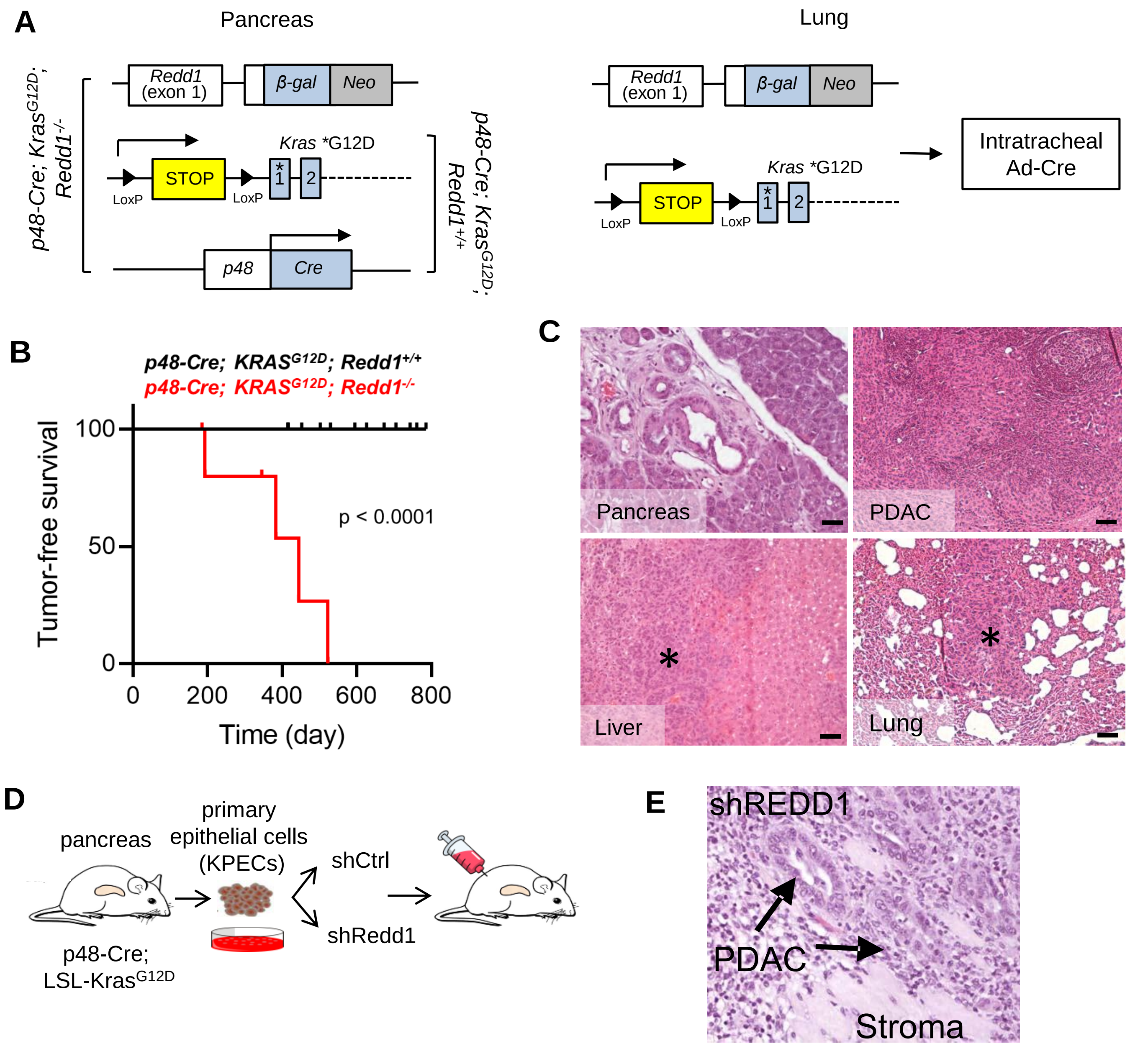

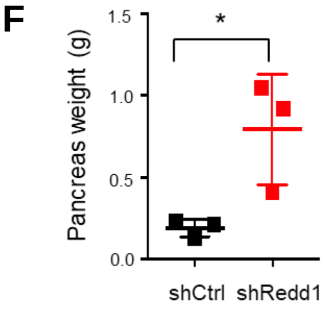

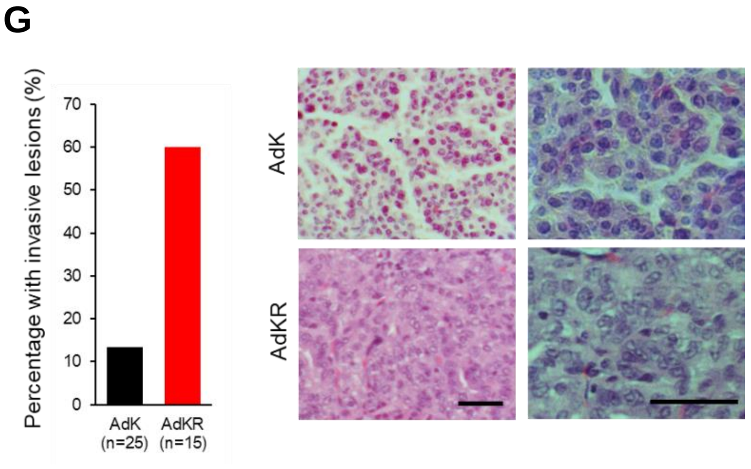

H

Lung	$Kras^{G12D}$; $Redd1^{+/+}$	$Kras^{G12D}$; $Redd1^{-/-}$
Metastasis	0/16	2/23

Qiao_Supplemental Figure 1, related to Figure 1.

(A) Schematic of genetic alleles employed. The *REDD1*$^{-/-}$ allele replaces the entire Redd1 coding region with the βgal/neo fusion cDNA.

(B) Tumor-free survival of pancreas cohort. Kaplan-Meier analysis of *p48-Cre;Kras*^{G12D}*;Redd*$^{-/-}$ *(p48KR)* mice (n = 6) and *p48-Cre;Kras*^{G12D}*;Redd1*$^{+/+}$ *(p48K)* mice (n = 12) based on the presence of invasive tumors (PDAC). Mice reaching euthanasia endpoint without PDAC were censored (tic marks). P value by log-rank test.

(C) Representative photomicrographs of H&E stained tissue sections showing metastases to liver and lung (indicated by *) from primary PDAC arising in *p48KR* mice. Scale bar, 100μm.

(D) Schematic representation of orthotopic pancreatic tumor model. Primary KRAS-activated pancreatic epithelial cells (KPECs) expressing control (shCtrl) or REDD1-deficient shRNA (shREDD1) are re-implanted into the pancreas of host mice.

(E) Representative H&E stained tissue sections of pancreas following orthotopic implantation, showing histology of poorly differentiated PDAC.

(F) Wet weight of mouse pancreas following orthotopic implantation of REDD1 knockdown or control primary KPECs. Horizontal lines indicate mean ± SD. * $p = 0.038$ by two tailed t-test.

(G) Analysis of the subset of *AdK* and *AdKR* mice whose lungs were prepped by perfusion at necropsy for whole-mount sectioning. Graph at left shows the percentage of lobes with invasive disease in the respective genotypes, as assessed with H+E (representative sections shown at right) and with elastic stain (not shown) by an expert lung pathologist (MMK). Pre-invasive lesions (*AdK*) are characterized by papillary architecture and condensed nuclei, while invasive lesions (*AdKR*) lack papillary morphology and have nuclei with open chromatin and prominent nucleoli. Scale bar, 250μm.

(H) Summary of metastases identified in the lung cancer cohort, by genotype.

Qiao_Supplemental Figure 2, related to Figure 2.

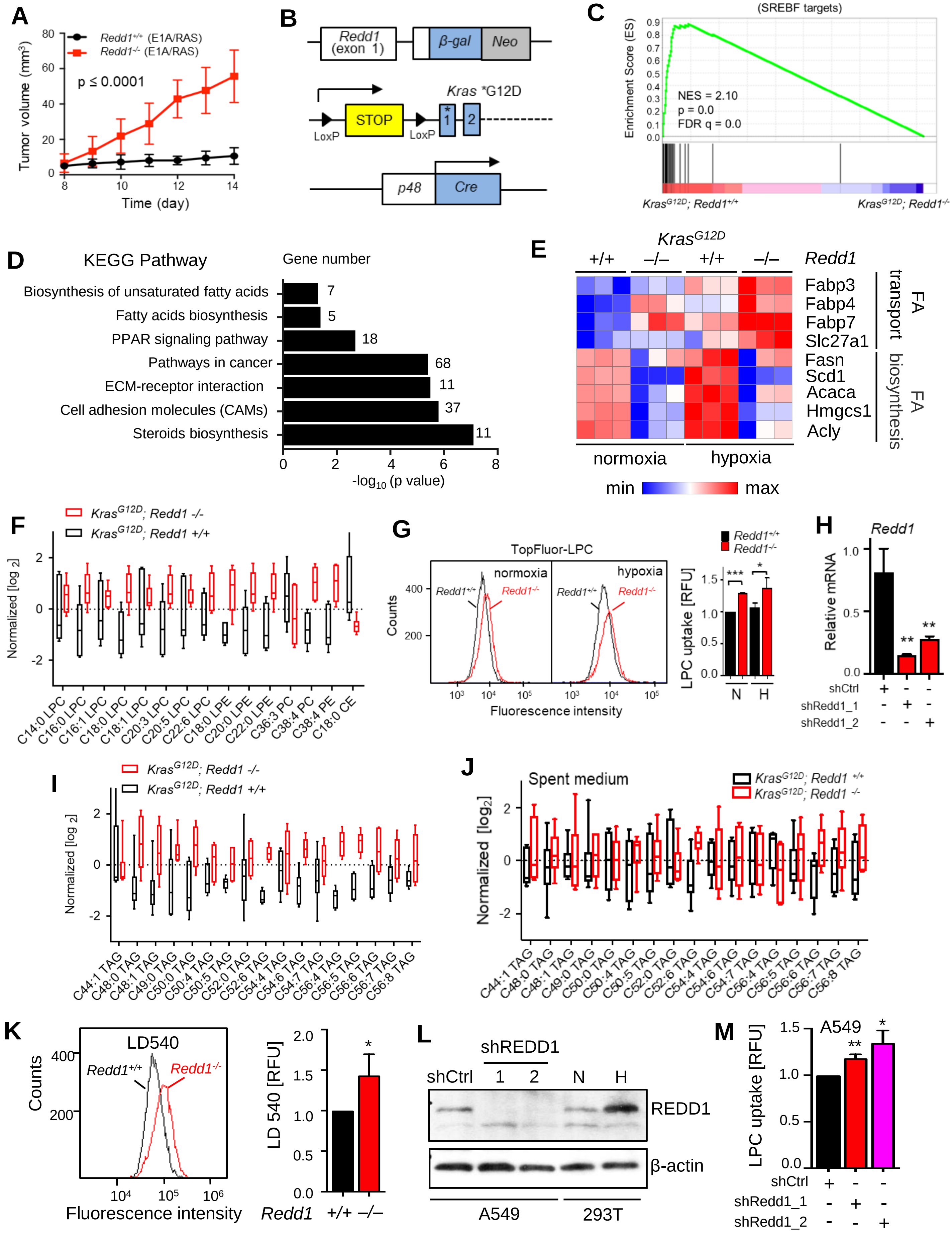

Qiao_Supplemental Figure 2, related to Figure 2.

(A) Loss of REDD1 confers tumorigenesis in the setting of mutant RAS expression. Primary *Redd1*$^{-/-}$ or *Redd1*$^{+/+}$ MEFs were co-infected with lentiviral constructs expressing the adenoviral E1A protein and KRAS G12V, then injected into immunodeficient (nude) mice. Primary *Redd1*$^{-/-}$ MEFs are non-tumorigenic with or without E1A expression alone (not shown). N=8 mice per genotype. Error bars denote SD. p-value by multiple measures ANOVA.

(B) Schematic of genetic alleles employed in Figure 2.

(C) GSEA plots of RNA-seq data showing suppression of "Horton SREBF targets" signature (systematic signature M3009) in paired primary *Kras*^{G12D}*;Redd1*$^{-/-}$ versus *Kras*^{G12D}*;Redd1*$^{+/+}$ primary cells, cultured under normoxia.

(D) DAVID analysis (https://david.ncifcrf.gov/) of KEGG pathway signatures, showing an unselected ranking by p-value of the top canonical pathways altered in RNA-seq data of *Kras*^{G12D}*;Redd1*$^{-/-}$ versus *Kras*^{G12D}*;Redd1*$^{+/+}$ primary MEFs (KRMEFs and KMEFs, respectively). Differential expression was defined as mean > two-fold difference in triplicate samples of each genotype when normalized by DeSeq. Cells from three mice per genotype were analyzed.

(E) Enrichment of lipid transport genes and suppression of de novo lipogenesis genes in KRMEFs as compared to KMEFs cultured under normoxia or hypoxia (1% O_2, 18 hr). RNA-seq data from three independent pairs of KMEFs and KRMEFs.

(F) Increased phospholipids in KRMEFs compared to KMEFs during growth under normoxia as detected by UHPLC-MS lipidomics analysis.

(G) Uptake of Top-Fluor-LPC in immortalized *Redd1*$^{-/-}$ as compared to *Redd1*$^{+/+}$ MEFs cultured under normoxia and hypoxia (1% O_2, 18 hr). Bar graph (right) represents quantification from three independent experiments. Error bars indicate SD.

(H) Knockdown of REDD1 in KRAS-activated primary pancreatic epithelial cells (KPECs) by lentiviral shRNA (shRedd1) or control vector (shCtrl), assessed by qRT-PCR analysis.

(I) TAGs enriched in KRMEFs under normoxia.

(J) TAGs enriched in KRMEFs under hypoxia remain unchanged in the corresponding culture medium as detected by lipidomics analysis. For (I) and (J), box-and-whisker plots denote normalized abundance of metabolite. Whiskers denote range and boxes denote SD. Triplicate samples from each of two mice per genotype were analyzed.

(K) Increased LD540 staining of neutral lipid droplets in in immortalized *Redd1*$^{-/-}$ MEFs as measured by flow cytometry analysis. Bar graph at right represents summary of three independent experiments from paired *Redd1*$^{+/+}$ and *Redd1*$^{-/-}$ MEFs.

(L) Immunoblot for REDD1 confirming shRNA knockdown of *REDD1* in A549 cells. Whole cell lysates from 293T cells cultured under normoxia (N) or hypoxia (H, 1% O_2, 4h) were used as positive control.

(M) Uptake of Top-Fluor-LPC in A549 cells with shCtrl or shREDD1 cultured under normoxia. Bar graph represents quantification from three independent experiments. Error bars indicate SD.
For all panels, * $p< 0.05$, ** $p < 0.01$.

Qiao_Supplemental Figure 3, related to Figure 3.

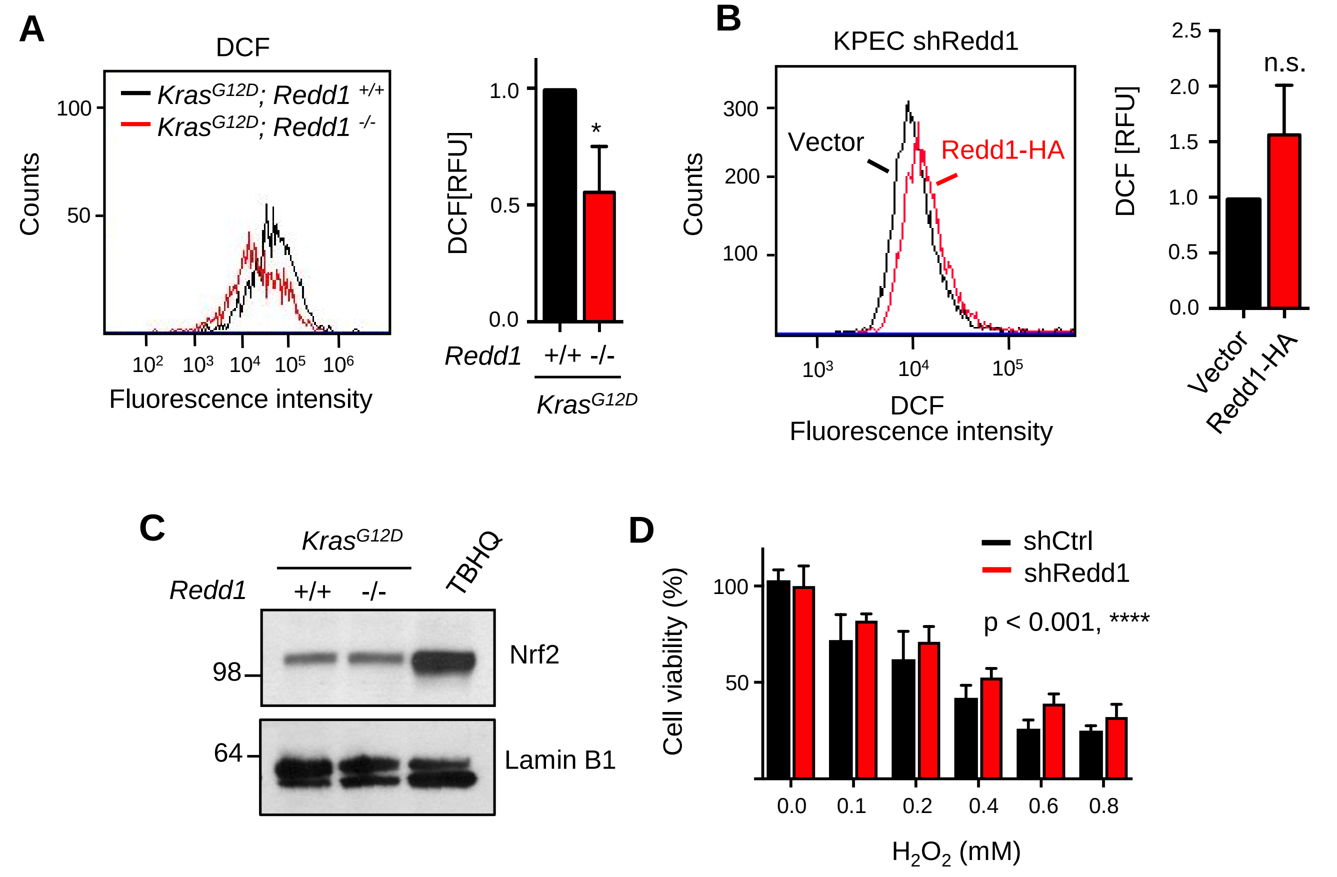

(A) Decreased ROS in KRMEFS as compared to KMEFs, assessed by staining with CM-H2DCFDA. Right: Summary from four independent experiments/mice measured in triplicate.

(B) Transfection of REDD1 induces H_2O_2 as compared to vector control in KPECs with stable knockdown of endogenous REDD1. Errors bars denote SD. Graph at right shows mean of two experiments. Error bars denote SD.

(C) Immunoblot showing equal NRF2 protein levels in nuclear extracts of KMEFs and KRMEFs. TBHQ (Tert-butylhydroquinone) treatment serves as a positive control for NRF2 induction.

(D) Ablation of REDD1 in primary KPECs induces resistance to oxidative stress (H_2O_2 treatment). Bars indicate mean of two experiments performed in duplicate. p-value by repeated measurements of ANOVA.

Unless otherwise noted, for all panels error bars denote SD.

Qiao_Supplemental Figure 4, related to Figure 4.

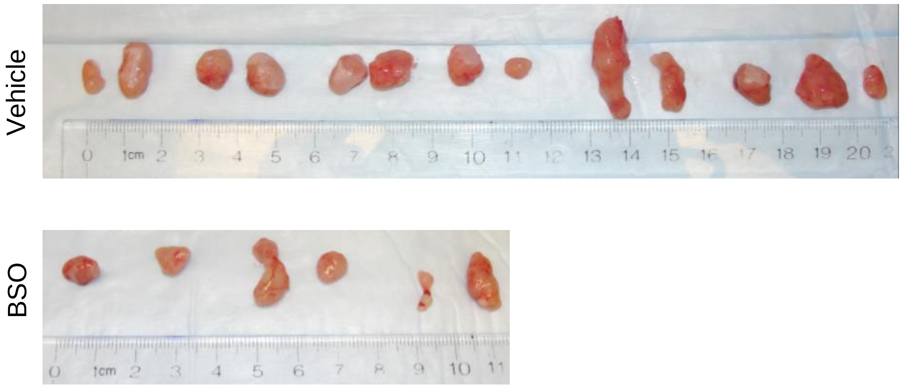

Kras^{G12D}*;Redd* $^{-/-}$ *(AdKR)* visible orthotopic tumors were harvested from mice treated with either vehicle or glutathione synthase inhibitor buthionine sulfoximine (BSO) at the end of the experiment.

Qiao_Supplemental Figure 5, related to Figure 5.

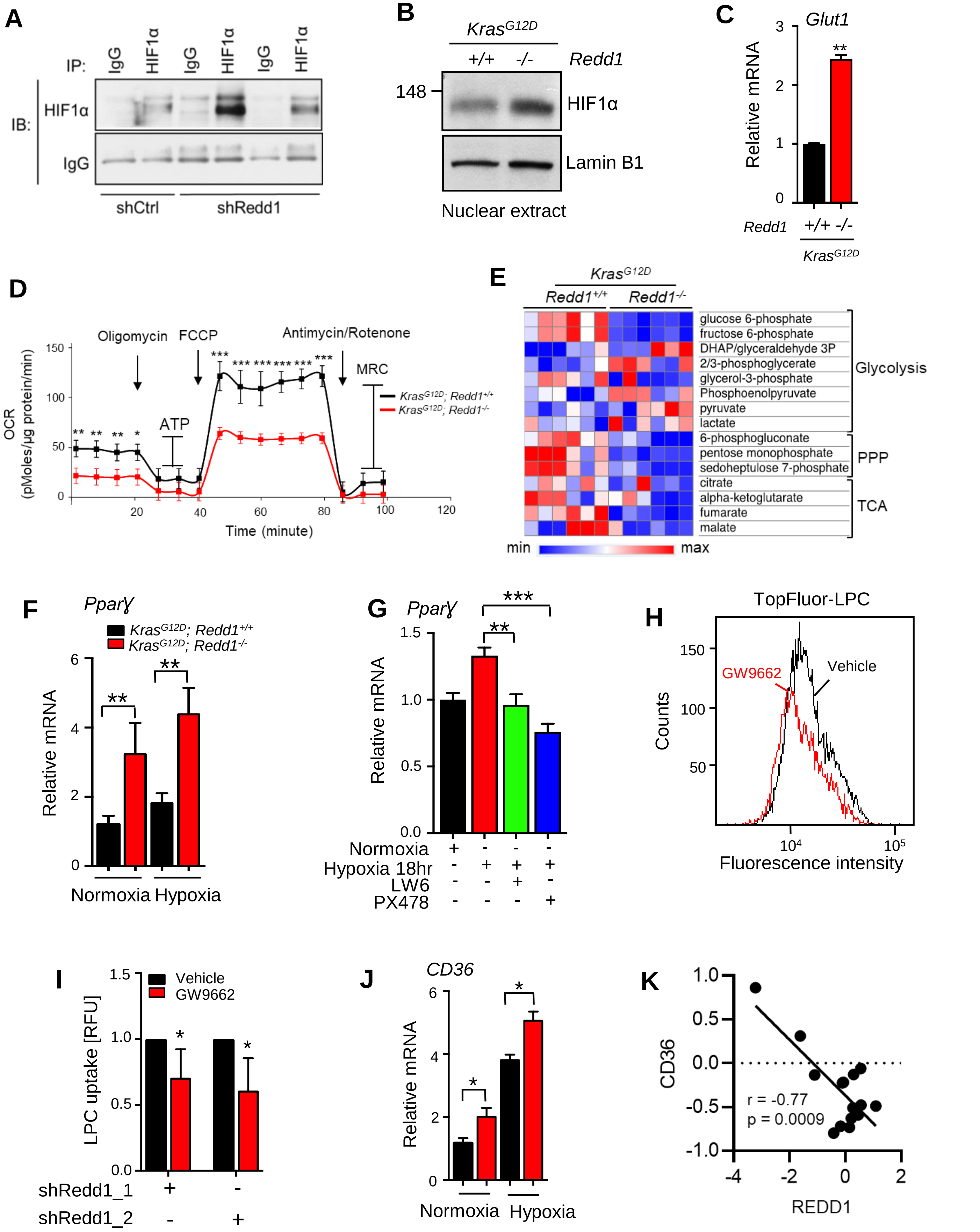

Qiao_Supplemental Figure 5, related to Figure 5.

(A) Knockdown of REDD1 in primary KPECs increases protein level of HIFα as detected by IP/Western analysis under hypoxic conditions (1% O2). IgG serves as a control for IP.

(B) Immunoblot detection of HIF1α protein level in nuclear extracts from paired primary KRMEFs and KMEFs (normoxia). Two independent experiments were performed.

(C) Expression of *Glut1* in KMEFs and KRMEFs, assessed by *q*RT-PCR analysis. Graph shows mean of three experiments. Error bars denote SD.

(D) Decreased basal O_2 consumption rate (OCR), mitochondrial ATP synthesis (ATP) and maximal respiratory capacity (MRC) in KRMEFs as measured via Seahorse XFe96. Shown is a representative experiment, performed three times.

(E) Heatmap depicting significantly altered glycolytic, PPP and TCA metabolite levels in KRMEFs versus KMEFs as analyzed by UHPLC-MS. Steady-state metabolomics data were normalized to sample median by MetaboAnalyst. PPP: Pentose phosphate pathway, TCA: tricarboxylic acid cycle. Triplicate samples from two mice per genotype were analyzed.

(F) Quantification of PPARγ expression levels in KRMEFs and KMEFs cultured under normoxia and hypoxia (1% O_2, 18 hr) as analyzed by RNA-seq analysis. n = 4 independent cultures for normoxia; n = 3 for hypoxia. Error bars denote SEM.

(G) Induction of PPARγ mRNA under hypoxia (1% O_2, 18 hr) in KPECs expressing a REDD1 shRNA can be suppressed by co-treatment with HIF1α inhibitors including LW6 (20μM) and PX478 (10μM), as assessed by *q*RT-PCR analysis. Data represent two experiments performed in duplicate.

(H) Topfluor-LPC uptake is inhibited by the PPARγ antagonist GW9662 treatment (20μM, 12 hr) in REDD1-ablated KPECs.

(I) Quantification of three independent experiments showing Topfluor-LPC uptake inhibited by GW9662 treatment as described in (H).

(J) Quantification of CD36 expression levels in KMEFs and KRMEFs cultured under normoxia and hypoxia (1% O_2, 18 hr), as analyzed by RNAseq analysis. n=4 independent cultures for normoxia; n=3 for hypoxia.

(K) CD36 expression is negatively correlated with REDD1 expression in Patient-Derived Xenograft (PDX) models of pancreas carcinoma. Values reflect tumor-intrinsic REDD1 levels, as non-tumor elements are largely murine-derived. Data were derived from the Mouse Models of Human Cancer Database (MMHC, formerly MTB), Mouse Genome Informatics, The Jackson Laboratory, Bar Harbor, Maine (http://tumor.informatics.jax.org/). Pearson r and p value (two-tailed) are shown.

Unless otherwise specified, error bars denote SD.

For all panels, * $p < 0.05$, ** $p < 0.01$, *** $p < 0.001$ by two-tailed t-test.

Qiao_Supplemental Figure 6, related to Figure 6.

A

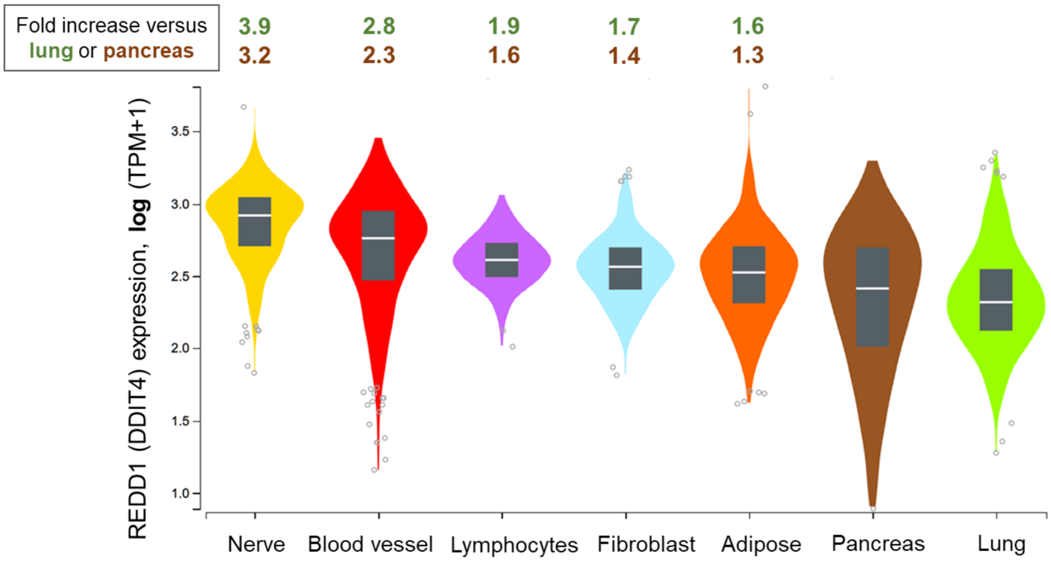

B

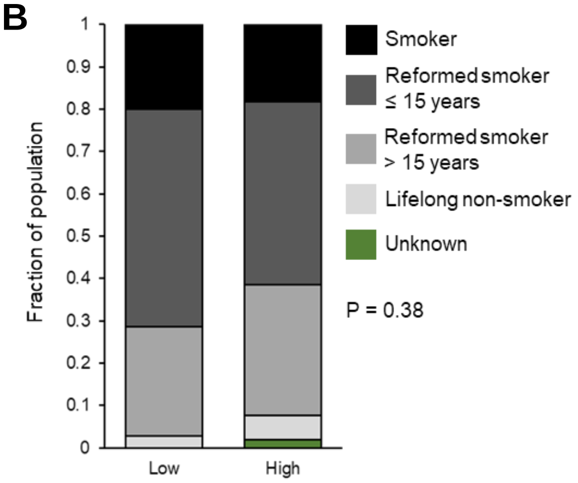

(A) REDD1 expression in normal human cells/tissues. Data were ranked from the highest (left) to the lowest (right) according to REDD1 expression. Mean fold-difference in expression versus normal lung or pancreas is shown at top. Data were obtained from the Genotype-Tissue Expression (GTEx) Project database (https://gtexportal.org/home/).

(B) Proportion of smokers with RAS-mutant lung adenocarcinoma is not significantly different based on REDD1 gene expression signature. Shown is the TCGA RAS-MUT LUAD population, stratified into top (high) and bottom (low) quartiles of the signature metagene values as in Fig. 6A. P value by chi-square test.

Analysis of REDD1 expression in human cells/tissues

Data for Supplemental Figure S6A were derived from The Genotype-Tissue Expression (GTEx) Project. The GTEx Project was supported by the Common Fund of the Office of the Director of the National Institutes of Health, and by NCI, NHGRI, NHLBI, NIDA, NIMH, and NINDS. The data used for the analyses were obtained from the GTEx Portal (https://gtexportal.org/home/) on 01/17/2020.

Generation of the REDD1-associated gene expression signature

Gene expression values were derived from RNA-Seq data for KRMEFs compared to KMEFs (4 vs. 4 samples). Reads were aligned to the mm10 reference genome with BWA by the MGH sequencing core. Bam to Sam file conversion, sorting, indexing, and file merging was done with SamTools (Li et al., 2009). FPKM values (Fragments per Kilobase of transcript Per Million mapped reads) were calculated by Cufflinks (Trapnell et al., 2010) version 2.2.1 suit of tools. First, cufflinks was used to normalize the results for the assembled isoforms using flags to normalize using only compatible hits that map to the transcriptome and only those in the upper quartile (and also masked chrM and rRNA). Second, cuffquant was run to quantize reads with rRNA masking. Finally, cuffnorm was run to produce normalized FPKM. Cufflinks FPKM was loaded into a matrix in R, quantile normalized, and then a variation filter was applied to remove genes with less than 1.5 fold minimum variation and 2 minimum absolute variation (leaving 4124 out of 23235 genes). A t-test was then performed to find genes significantly varying between KRMEF and KMEF and corrected for multiple hypothesis testing

using the Benjamini-Hochberg (Benjamini & Hochberg, 1995) step-up FDR-controlling procedure. Genes from the KRMEFs vs. KMEFs with a p-value less than 0.05 were selected leaving 415 genes (197 up in KRAS null and 218 down in KRAS null), then X and Y chromosome genes were removed from the signatures leaving 187 up and 210 down genes, which were used to make a meta-gene to analyze the signature in patient tumor samples. These genes were mapped to genes in TCGA RNASeqV2 data leaving 159 genes up and 180 genes down. The meta-gene was made from mean of the log2 of KRAS null up genes thresholded to a minimum of 0.1 minus the mean of the log2 of KRAS null down up genes thresholded to a minimum of 0.1.

Qiao_Supplemental References.

Benjamini Y, Hochberg Y. 1995. Controlling the False Discovery Rate: a Practical and Powerful Approach to Multiple Testing. *Journal of the Royal Statistical Society* **57**: 11.

Li H, Handsaker B, Wysoker A, Fennell T, Ruan J, Homer N, Marth G, Abecasis G, Durbin R, Genome Project Data Processing S. 2009. The Sequence Alignment/Map format and SAMtools. *Bioinformatics* **25**: 2078-2079.

Trapnell C, Williams BA, Pertea G, Mortazavi A, Kwan G, van Baren MJ, Salzberg SL, Wold BJ, Pachter L. 2010. Transcript assembly and quantification by RNA-Seq reveals unannotated transcripts and isoform switching during cell differentiation. *Nat Biotechnol* **28**: 511-515.
